# Extensive Proteomic Approach to Assess Protein Alterations in Human Hepatoma Huh7 Cells Infected with West Nile Virus

**DOI:** 10.3390/ijms27209387

**Published:** 2026-10-21

**Authors:** Enxhi Shaba, Claudia Landi, Ilaria Vicenti, Lorenza Vantaggiato, Francesco Saladini, Camilla Biba, Niccolò Bartolini, Maurizio Zazzi, Luca Bini

**Affiliations:** 1 Functional Proteomics Lab, Department of Life Sciences, University of Siena, 53100 Siena, Italy; enxhi.shaba@unisi.it (E.S.); lorenz.vantaggiato2@unisi.it (L.V.); luca.bini@unisi.it (L.B.); 2 Medical Biotechnologies Department, University of Siena, 53100 Siena, Italy; vicenti@unisi.it (I.V.); saladini6@unisi.it (F.S.); camilla.biba@student.unisi.it (C.B.); niccolo.bartolini@student.unisi.it (N.B.); maurizio.zazzi@gmail.com (M.Z.); 3 Departmental Faculty, UniCamillus, Saint Camillus International University of Health Sciences, Via di Sant’Alessandro, 8, 00131 Rome, Italy

**Keywords:** extensive proteomics, West Nile Virus, ECM remodeling, unfolded protein response, iron homeostasis, One Health

## Abstract

West Nile Virus (WNV) is a significant global health threat, and the molecular mechanisms of its pathogenic activity in human hosts remain partially understood. This study applies an extensive proteomic approach combining “top-down” (2DE/MS) and “bottom-up” (LC-MS/MS) technologies to investigate protein changes in the Huh7 human hepatoma cellular model at 48 h post-infection with WNV. Our results demonstrate that WNV infection induces a massive downregulation of host proteins, particularly those involved in cell–cell adhesion and extracellular matrix (ECM) integrity (FN1, VTN, desmosome proteins and tight junctional proteins), which supports theories of blood–brain barrier disruption. Moreover, we highlighted a modulation of iron homeostasis (FTH1, IRP1, and IREB2) reflecting a “nutritional immunity” host response aimed at limiting host enzyme functions essential for virus replication. In particular, the top-down approach demonstrated a significant protein species modulation related to the unfolded protein response (UPR) (HSP90, HSPA8), as well as the hijacking of translation initiation factors (EIF1, eIF3 complex, and EIF4A1), which suggests viral strategies for immune evasion and efficient replication. Taken together, these findings offer a comprehensive overview of WNV-induced perturbations, revealing the quantitative modulation of proteins and potential post-translational signatures, thereby providing a robust foundation for identifying new antiviral drug targets and therapeutic strategies.

## 1. Introduction

West Nile Virus (WNV) is a member of the *Flaviviridae* family, together with other widely known pathogens, such as the dengue (DENV), Zika (ZIKV), Japanese Encephalitis (JEV) and yellow fever (YFV) viruses [1]. To date, WNV infection represents a global health challenge, as rising temperatures and increased rainfall, due to climate change, are contributing to the widespread diffusion of mosquito vectors [2]. WNV’s main route of infection is via mosquitoes, and its main host reservoirs are birds; human, horses and other vertebrate animals can be infected but are defined as “dead-end” hosts. Most human infections remain asymptomatic or develop mild flu-like symptoms. Only <1% of infected subjects develop neuroinvasive disease, which is sufficient to make WNV an important causative agent of human viral encephalitis worldwide [3,4]. WNV is characterized by an icosahedral nucleocapsid surrounded by a lipid envelope and contains a single-stranded positive-sense RNA genome encoding a single open reading frame. This is translated into a polyprotein which is then cleaved by viral and host proteases to generate ten functional viral proteins. The viral proteins include three structural proteins (capsid C, PrM protein, and envelope E), and seven non-structural proteins (NS1, NS2A, NS2B, NS3, NS4A, NS4B and NS5), exerting multiple functions in viral replication and the evasion of host cell defenses [5,6]. Indeed, WNV has developed several strategies to counteract and evade the immune response, which allows the virus to persist efficiently in the host and favors viral dissemination and central nervous system (CNS) invasion [7] through incompletely understood mechanisms. Possible hypotheses include WNV’s ability to cross the blood–brain barrier (BBB) through its tight junctions, the direct infection of endothelial cells in the cerebral microvasculature, transport by infected leukocytes, and/or direct axonal retrograde transport from peripheral neurons [8]. Despite extensive efforts over the last 20 years to develop effective therapeutic and preventive strategies, no specific antiviral treatments or approved human vaccines are available for humans, and only supportive care is available [5]. Thus, a deeper understanding of WNV pathogenesis may provide new potential antiviral drug targets. This urgent need fits perfectly in the concept of “One Health”, according to which the health of people, animals and ecosystems are tightly interconnected. Indeed, understanding and managing zoonotic pathogens starting from their animal source represents the most effective strategy for avoiding human and animal infections [9]. From this perspective, omics sciences represent a powerful tool to investigate how the pathogen interacts with and infects host cells, including the host cell conditions that may facilitate or counteract the infection, how the host immune system activates and acts, how the pathogen replicates within host cells and if it has developed strategies to avoid cellular responses against the pathogen itself. In particular, proteomic-based technologies have been increasingly used to provide new insights into host–pathogen interaction [10]. In fact, mass spectrometry (MS)-based proteomics has gained global interest for its application in RNA virus biology due to its potential in shedding light on virus–host interactions and perturbations and helping to define antiviral targets [11]. Indeed, proteomics can provide a snapshot of a specific pathophysiological condition of an organism by analyzing the entire protein content of a cell/tissue/organ at any one time, considering also all the protein species generated by all the post-translational modifications (PTMs) that a protein may acquire. Proteomics can be divided into two main approaches: “bottom-up”, mainly oriented toward defining and quantifying the total protein contents of a sample, and “top-down”, useful for studying protein species [12,13,14,15]. To benefit from the potential of each technology, we combined bottom-up and top-down proteomics in a so-called “extensive proteomic approach”, increasing the possibility of defining and quantifying the total protein content, including protein species. Therefore, our study attempted to provide a wider overview of human cellular reactions to WNV infection through the application of a two-dimensional gel electrophoresis (2DE)/mass spectrometry (MS)-based approach (top-down) and a liquid chromatography–tandem mass spectrometry (LC-MS/MS)-based approach (bottom-up) to the same samples of uninfected and WNV-infected human hepatoma cells (Huh7). This cell line provides a relevant physiological human hepatic context and supports WNV replication with early cytopathic effects; thus, it is highly preferable to the similar, widely used VERO cells [16]. To the best of our knowledge, this study is the first extensive proteomic investigation on human cells infected with WNV using a combination of top-down and bottom-up approaches.

## 2. Results

### 2.1. Experimental Design: An Extensive Proteomic Approach

**Figure 1:**
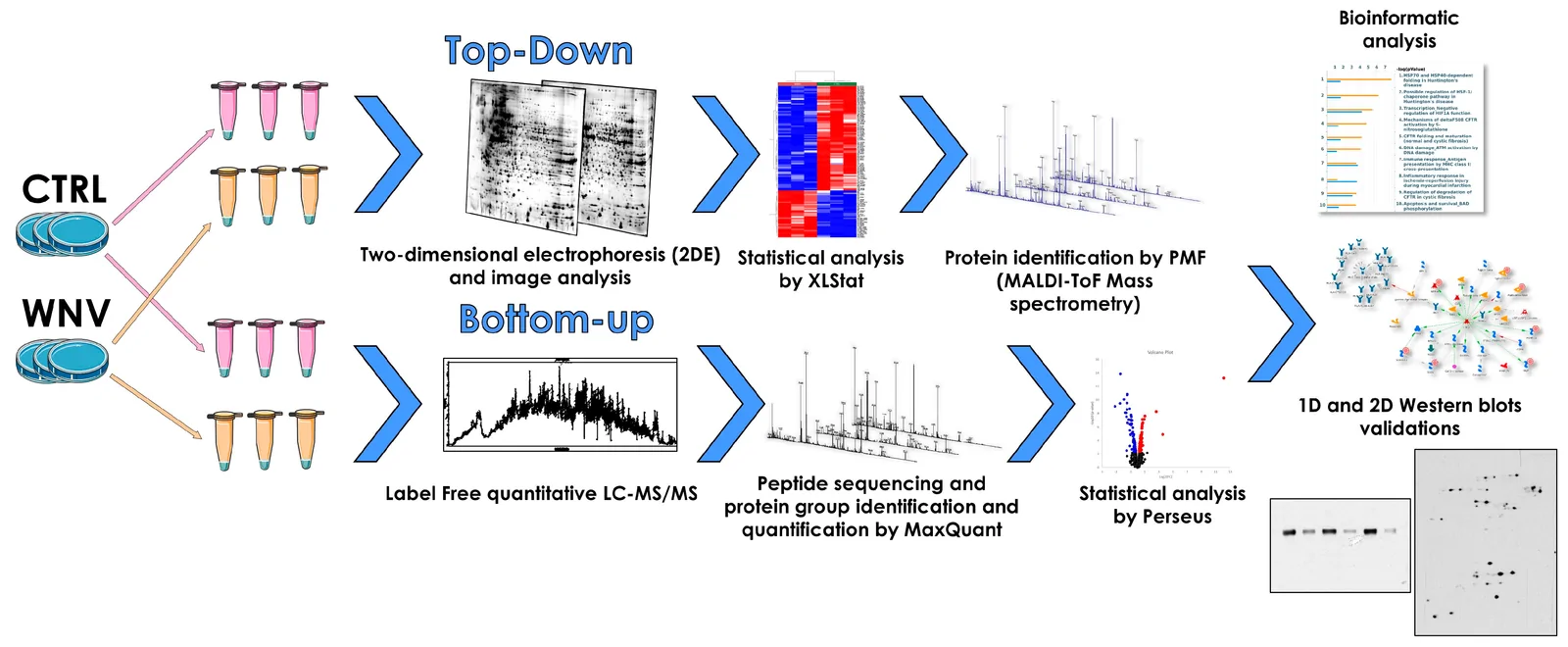
Experimental design.

Given the very little information about how human cells respond at the protein level once infected by WNV, and specifically regarding the main molecular mechanisms that are modulated by WNV viral activities, our study aimed to provide a comprehensive proteomic evaluation of the human cellular response to WNV infection through the application of the two different proteomic approaches: a gel-based proteomic analysis as a top-down approach and a gel-free proteomic analysis as a bottom-up approach. For this purpose, two parallel proteomic analyses were carried out, starting from the same infected (WNV) and uninfected (CTRL) Huh7 cell line. The applied experimental design, as described in Figure 1, consisted of three biological replicates per condition, CTRL and WNV, analyzed at 48 h post-infection (Multiplicity of Infection, MOI 0.005) (Table S1). To reduce experimental variability and ensure a correct and robust comparison between the two approaches, each biological replicate per condition was split into two, intended for two different proteomic preparations and procedures: top-down and bottom-up approaches (Figure 1). In the top-down approach, the extracted whole protein species were resolved by high-resolution 2DE, with all PTMs preserved; later, 2DE gels were examined by image analysis, and differential spots within specific statistical inclusion criteria were identified by MALDI-ToF MS (Bruker Daltonics, Bremen, Germany) using Peptide Mass Fingerprinting (PMF). At this step, the identified differential protein species were subjected to bioinformatic analysis, where the most enriched molecular pathways were highlighted. On the other hand, the bottom-up approach included a label-free quantitative (LFQ) LC-MS/MS analysis (LC-MS/MS system consisting of an EXPLORIS 240 Orbitrap mass spectrometer coupled to a Vanquish Neo UHPLC, Thermo Scientific, Waltham, MA, USA), in which extracted proteins were first digested by a specific proteolytic enzyme, and then peptides were separated by HPLC and analyzed by a highly sensitive tandem mass spectrometer to enable protein identification and quantification. Then, the identified proteins were statistically analyzed, and the differential proteomic data set was functionally investigated by bioinformatics.

### 2.2. Top-Down Differential Proteomic Analysis

**Figure 2:**
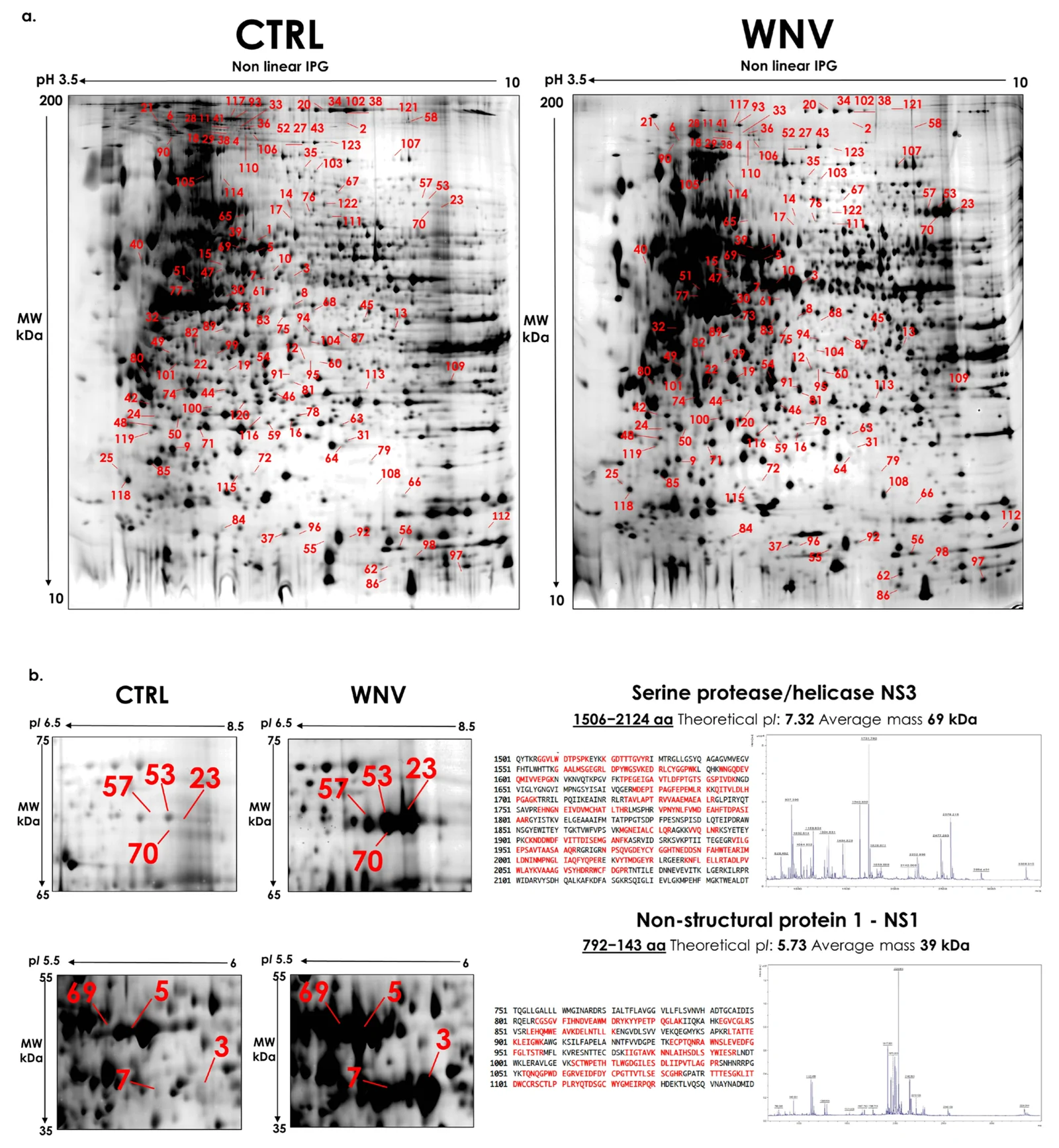
Top-down proteomic analysis. (**a**) Representative 2DE electropherograms of CTRL vs. WNV-infected Huh7 cells. 2DE gels (*n* = 3 per condition) show an average of 3000 spots, and the differentially abundant spots between the two considered conditions (CTRL at left; WNV at right) are reported in red (one-way ANOVA test; *p*-value ≤ 0.05; fold change ≥ 1.8). Spot numbers correspond to those shown in Table S2. (**b**) Resolution by 2DE and MS identification of WNV NS3 and NS1 protein species. For each protein, NS1 and NS3, spot protein species are shown in representative CTRL and WNV 2DE gels (spot numbers marked in red and correspond to those shown in Table S2) on the right, as well as protein name, aminoacidic residues, isoelectric point and molecular weight, protein sequence (matched peptides are marked in red) and representative MALDI-ToF mass spectrum on the left.

CTRL and WNV protein extracts were resolved by 2DE, which enabled the observation of all protein species with an isoelectric point (p*I*) between 3 and 10 and a molecular weight (MW) between 200 and 10 kDa (Figure 2a). An average of 3060 spots were detected in 2DE gels by image analysis. An ANOVA test highlighted 122 differentially abundant spots (*p*-value ≤ 0.05 and fold change ≥ 1.8) (Figure 2a and Table S2). Protein identification by PMF was successful in identifying 107 protein species (annotated spectra and mass lists are provided as Supplementary Materials). Among the more abundant spots in the WNV samples, WNV viral protein species were identified (Figure 2b): four protein species of serine protease/helicase NS3 (1506-2124 aa residues in viral polyprotein; theoretical p*I* 7.32; average mass 69 kDa) were identified from spots 23, 53, 57 and 70, with a p*I* between 6.5 and 8.5 and an MW between 75 and 65 kDa; mass lists of tryptic peptides of these spots covered most of the NS3 sequence, specifically from residue 1506 to 2074. In addition, four protein species of NS1 (792-1143 aa residues in viral polyprotein; theoretical p*I* 5.73; average mass 39 kDa) were identified from spots 3, 5, 7 and 69, with a p*I* between 5.5 and 6 and an MW between 55 and 35 kDa; mass lists of tryptic peptides of these spots covered most of the NS1 sequence, specifically from residue 806 to 1130.

**Figure 3:**
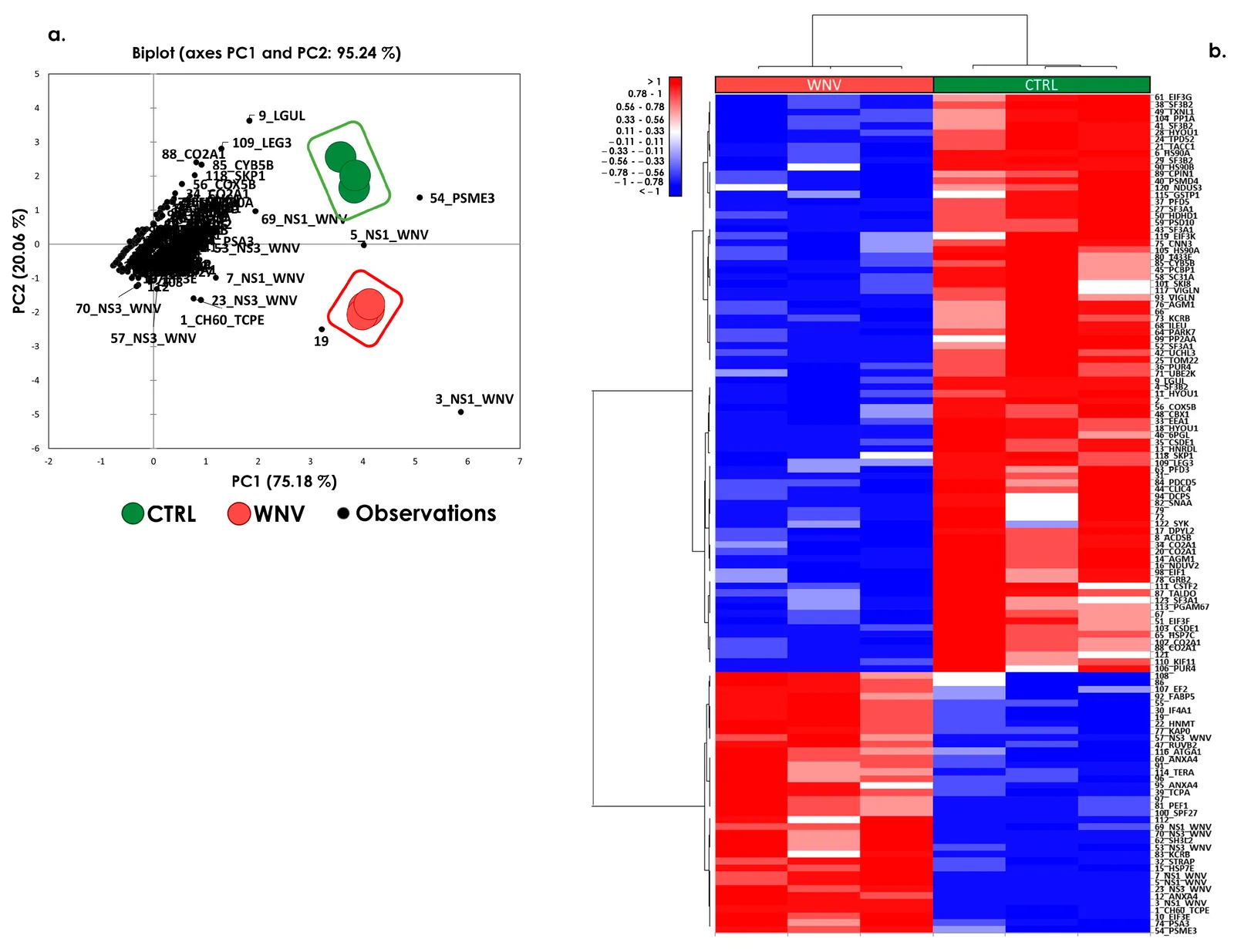
Multivariate analysis of top-down differential proteomic data. (**a**) Principal Component Analysis (PCA) of the differential spots obtained by 2DE comparison of CTRL (*n* = 3) and WNV-infected (*n* = 3) cells. PCA summarizes 75.18% of variance. Differential protein spots are marked as black spots. CTRL samples are colored in green, while WNV samples are colored in red. (**b**) Heatmap analysis of the differential spots obtained from CTRL vs. WNV comparison. High-abundance spots are reported in red, while low-abundance ones are reported in blue.

Principal component analysis (PCA) of the top-down differential data (Figure 3a) showed that the differential proteomic profile of the CTRL vs. WNV samples well characterizes the cellular changes after WNV infection, as the samples separate well along principal component 2 and tightly group within the same CTRL or WNV condition. Indeed, the presence of spots of viral proteins NS1 and NS3 in the WNV samples, as well as the increased abundance of spot 19 (not successfully identified by MS) and of spot 54 of the proteasome activator complex subunit 3 (PSME3), showed a greater relevance in control/infection discrimination. The heatmap analysis in Figure 3b provides evidence of the similar behavior of differential protein species within the same condition, CTRL or WNV, as samples of the same condition cluster together; moreover, the heatmap highlights two major groups of differential protein species, one composed of 38 more abundant protein species in WNV with respect to CTRL and 84 less abundant protein species in WNV compared with CTRL (Figure 3b and Table S2). Among the first group, NS1 and NS3 viral protein species can be observed (Figure 3b).

**Figure 4:**
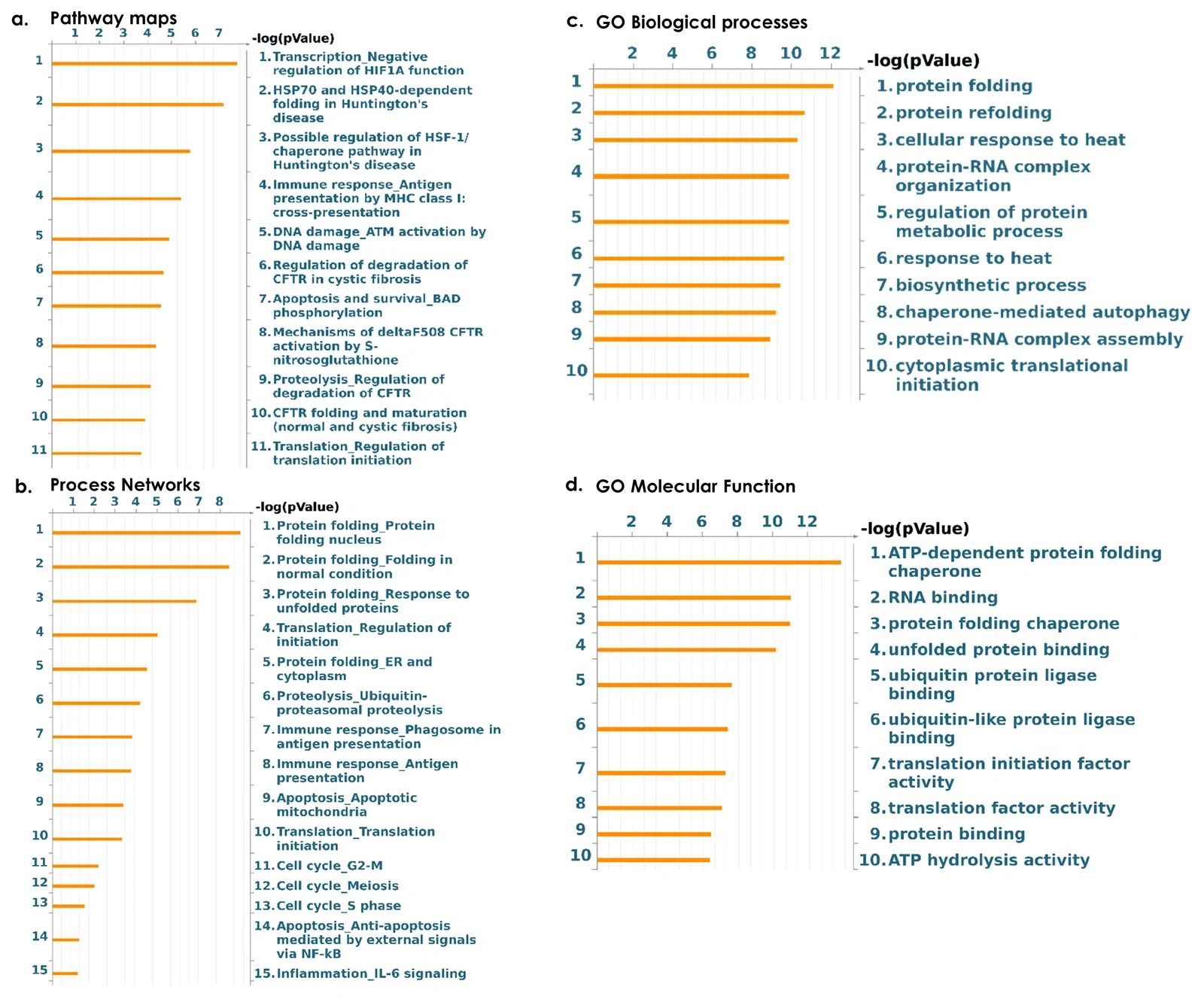
Enrichment analysis of top-down differential proteomic data. Enrichment analysis via pathway maps analysis (**a**), process networks (**b**), GO biological processes (**c**) and GO molecular function (**d**) by MetaCore software.

Pathway analysis of the top-down differential protein species evidenced the modulation of interesting molecular mechanisms by WNV infection via the suggestion of particular well-known, enriched pathways (Figure 4a): the negative regulation of HIF1-alpha function and possible regulation of the HSF-1/chaperone pathway; HSP70 and HSP40-dependent protein folding and regulation of protein degradation; antigen presentation by MHC class I; ATM activation by DNA damage; apoptosis and survival; and regulation of translation initiation. Indeed, enrichment analyses by process networks, GO biological processes and GO molecular function confirmed the modulation of the following mechanisms: protein folding either in support of de novo proteins or as a stress response to unfolded proteins, regulation of translation initiation and RNA binding, ubiquitin-proteasomal proteolysis, immune response via antigen presentation, inflammatory response, apoptosis and cell cycle regulation (Figure 4b–d).

### 2.3. Bottom-Up Differential Proteomic Analysis

LC-MS/MS runs included three technical replicates for each of the three biological replicates in the CTRL and WNV conditions. Tandem MS analysis resulted in the identification of 3356 proteins, with a total count of 36,981 peptides. Protein identifications were filtered out for reverse and contaminants, which resulted in 3293, with 36,897 peptides counted (proteins/peptides identification and quantification data are provided in the “combined” folder and are available within the data set identifier PXD080593 uploaded via the PRIDE partner repository). Among these, 2835 proteins were correctly quantified, as 70% of valid LFQ values within at least one group (CTRL or WNV) was the minimum inclusion criterion. Multivariate analysis and quality control analysis were performed, confirming the robustness and high quality of the obtained protein identifications and LFQ values (Figure S1). Statistical analysis by Student’s *t*-test was performed, taking into account biological triplicates, and it highlighted 797 differential proteins between CTRL and WNV (FDR 0.05), of which 127 had a fold change ≥ 1.8 and were considered for further analysis (Table S3).

LFQ LC-MS/MS analysis resulted in the confirmed identification and quantification of WNV genome polyprotein (ID 118) only in WNV samples (Table S3). Furthermore, Figure S2 shows that all 10 viral proteins were identified (covered sequences are marked in red) with a sequence coverage of at least 55% on average: capsid protein C; protein prM; envelope protein E; non-structural proteins NS1, NS2A, NS2B serine protease/helicase NS3, NS4A, NS4B; and RNA-dependent RNA polymerase NS5.

**Figure 5:**
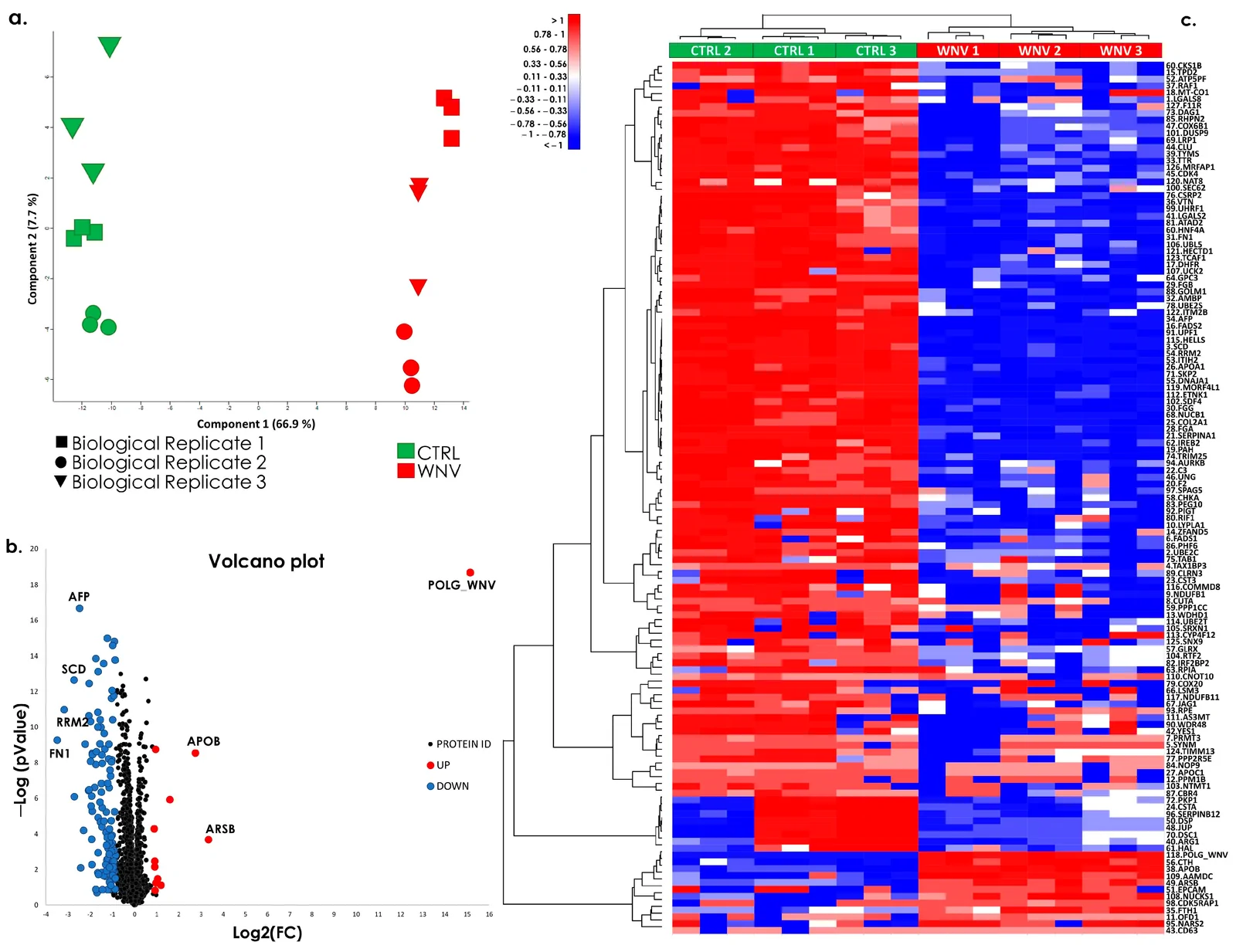
Multivariate analysis of bottom-up differential proteomic data. (**a**) Principal Component Analysis (PCA) of the differential spots obtained by label-free quantitative LC-MS/MS comparing CTRL (*n* = 3) and WNV-infected (*n* = 3) cells by Student’s t-test statistical analysis, with permutation-based FDR application. PCA is composed of PC1, which summarizes 66.9% of variance, and PC2, which summarizes 7.7% of variance. CTRL samples are colored in green, while WNV samples are colored in red. Biological replicates are represented by different shapes. (**b**) Volcano plot of differential bottom-up proteomic data between the CTRL and WNV-infected samples with FDR ≤0.05 and a fold change ≥ 1.8. Upregulated proteins in WNV samples are marked with red, while downregulated proteins in WNV samples are marked in blue. (**c**) Heatmap analysis of the differential bottom-up proteomic data. High-abundance proteins are reported in red, while low-abundance ones are reported in blue. CTRL samples are colored in green, while WNV ones are colored in red.

PCA performed on differential proteins showed that the differential proteomic profile clearly distinguishes the uninfected and WNV-infected conditions (Figure 5a). In addition, volcano plot and heatmap analysis provided evidence that WNV infection determines an overall downregulation of 115 proteins, compared to 12 upregulated proteins, still after the infection (Figure 5b,c). Among the upregulated proteins, the WNV genome polyprotein (GP1_WNV) can be observed as a qualitative difference.

**Figure 6:**
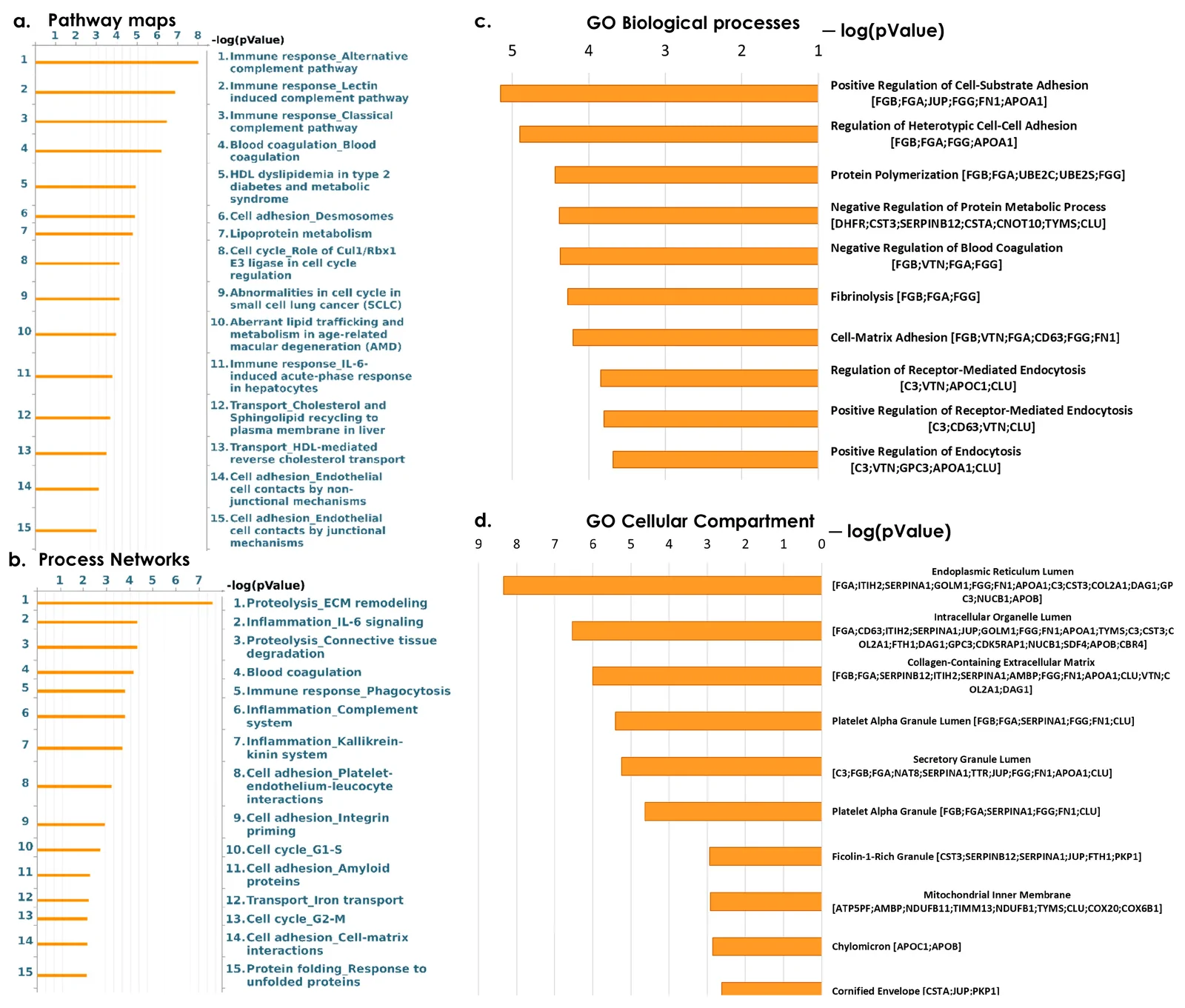
Enrichment analysis of bottom-up differential proteomic data. Enrichment analysis conducted via pathway maps (**a**) and process networks (**b**) analysis by MetaCore software; enrichment analysis conducted via GO biological processes (**c**) and GO molecular function (**d**) analysis by EnrichR.

Following the submission of the bottom-up differential proteomic data to bioinformatic tools, particular molecular mechanisms were observed as being enriched, highlighting different aspects of the cellular response to the infection. Indeed, pathway analysis (Figure 6a) reported the significant modulation of the activation of the immune response via the complement pathway and via IL-6, the cell cycle, the dysregulation of cell adhesion (desmosomes, endothelial cell contacts by junctional and non-junctional mechanisms), lipoprotein metabolism (aberrant lipid trafficking, cholesterol and sphingolipid recycling to plasma membrane), cell cycle regulation, and blood coagulation. Enrichment analyses by process networks and GO biological processes (Figure 6b,c) confirmed, indeed, the involvement of specific mechanisms: cell–cell and cell–substrate adhesions (platelet–endothelium–leucocyte interactions, integrins), cell–matrix interactions and extracellular matrix (ECM) remodeling (connective tissue degradation); iron transport; inflammation via the complement system, kallikrein–kinin system, IL-6, and the immune response by phagocytosis; cell cycle regulation at the G1-S and G2-M phases; unfolded protein response (UPR) and regulation of protein metabolic processes; regulation of blood coagulation and fibrinolysis; and regulation of endocytosis. Moreover, enrichment analysis by GO cellular compartment (Figure 6d) highlighted the most involved cellular sections: endoplasmic reticulum, collagen-containing ECM, secretory granule lumen, mitochondrial inner membrane, lipoproteins and lipid envelope.

### 2.4. Extended Bioinformatic Analysis

**Figure 7:**
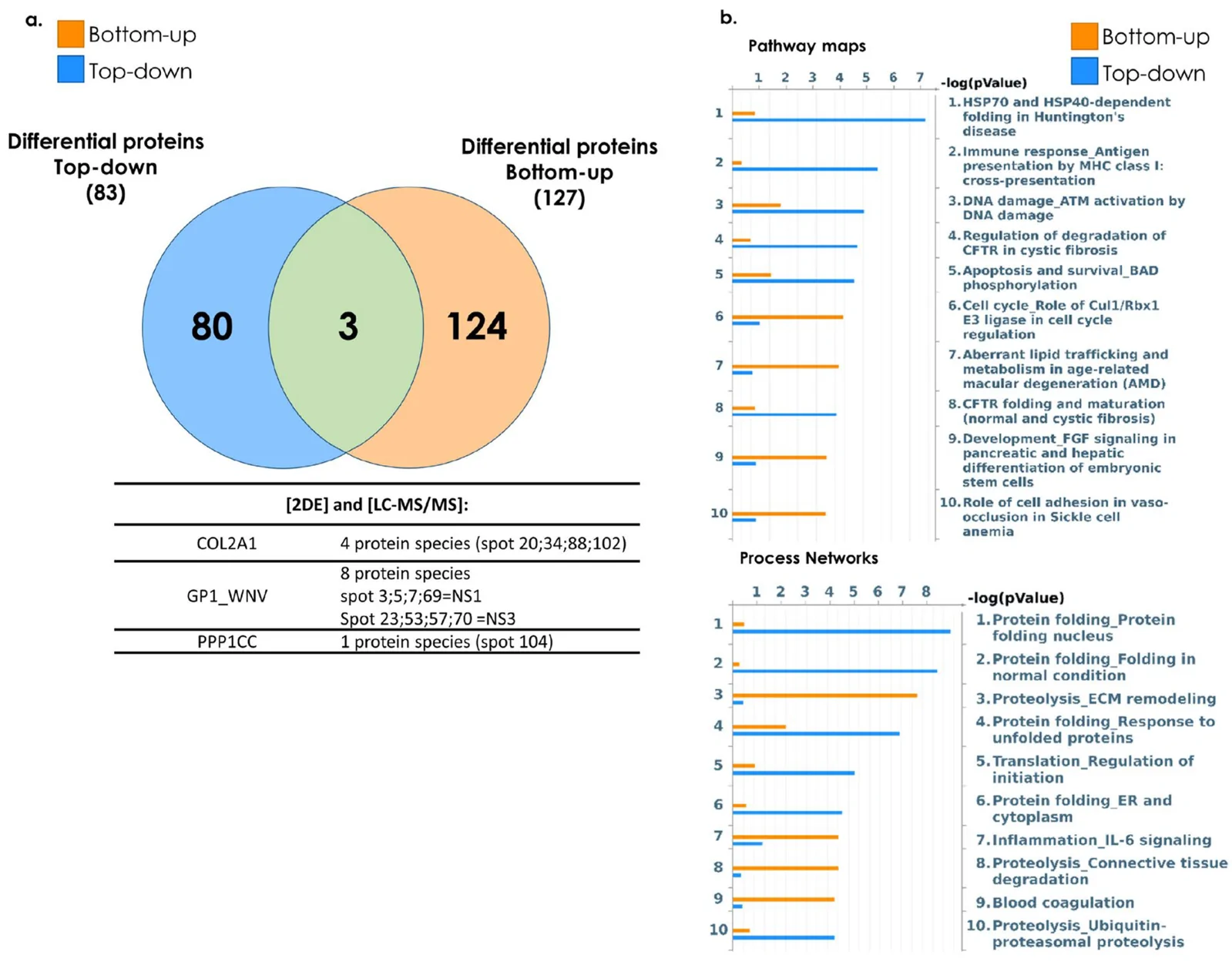
Extensive proteomic approach. (**a**) Venn diagram of top-down-identified differential proteins and differential proteins by bottom-up approach (with *p*-value ≤ 0.05 and FC ≥ 1.8). Below, table of common differential proteins indicated by their gene names and number of protein species (spot numbers corresponding to numbers in Table S2) in top-down results. (**b**) Comparative enrichment analysis of top-down (blue bars) and bottom-up (orange bars) differential proteomic data sets via pathways maps and process networks by MetaCore software.

Once the proteomic data sets from the top-down or bottom-up approaches were investigated independently, a combined analysis considering both data sets was performed. First, most of the top-down differential protein species were also identified by the bottom-up approach, but no quantitative variation in the total proteins of most of them was observed. When a stringent inclusion criterion was applied (FDR 0.05, fold change ≥ 1.8), the common differential proteins were three: collagen alpha-1(II) chain (COL2A1), GP1 WNV polyprotein and serine/threonine-protein phosphatase PP1-gamma catalytic subunit (PPP1CC) (Figure 7a). Of note, the 80 differential proteins exclusive to the top-down approach varied in abundance just for one to three protein species, and not for the total proteins, which reinforces the idea that an extensive proteomic approach could be useful for defining different sets of proteins influenced by WNV infection in human cells also at a post-translational level. A comparative enrichment analysis of the two data sets (Figure 7b) highlighted the same common modulated pathways, shedding light on the ability of each approach to provide reliable and robust results; however, each data set contributes to some of them to a different extent, which justifies the choice of a combination of the two proteomic approaches to obtain a more comprehensive view of the cellular response at the proteomic level. Specifically, the top-down data set contributed more significantly to enriched pathways such as protein folding under normal conditions and the UPR, antigen presentation, ubiquitin-proteasomal proteolysis, DNA damage response via ATM activation and regulation of translation initiation. On the other hand, pathways more strongly associated with the bottom-up differential proteomic data are ECM remodeling, cell adhesions and cell–matrix interactions, cell cycle regulation, inflammation via IL-6, blood coagulation, and lipid trafficking and metabolism. Furthermore, both proteomic data sets were subjected to bioinformatic analysis by Pathogen–Host Interactome Prediction using structure similarity (PPI P-HIPster), which evidenced some specific differential proteins which might be potential interactors of WNV viral proteins (Table S4): among top-down differential proteins, 14-3-3 protein epsilon (YWHAE), Heat shock cognate 71 kDa protein (HSPA8), Heat shock protein HSP 90-alpha and -beta (HSP90AA1, HSP90AB1), 26S proteasome non-ATPase regulatory subunit 4 (PSMD4), proteasome activator complex subunit 3 (PSME3) and growth factor receptor-bound protein 2 (GRB2) might hypothetically interact with envelope protein E, as well as capsid protein C and protein prM (polyprotein 1-791 aa) and non-structural proteins NS1, NS2A, NS2B, NS3 and NS4 (polyprotein 1-2273 aa); protein AMBP (AMBP) and fibronectin (FN1), as bottom-up differential proteins, are defined as hypothetical interactors of NS3.

### 2.5. The Unfolded Protein Response, ECM Remodeling and Iron Transport Modulation Induced by WNV Infection: Western Blot (WB) Validations

This extensive proteomic approach demonstrated the dysregulation of specific molecular mechanisms after WNV infection, both considering top-down and bottom-up proteomic data as independent or complementary sources. Here, we used a mono-dimensional (1D) or a bi-dimensional (2D) Western blot approach to validate candidate molecules that are key elements of these specific molecular mechanisms.

#### 2.5.1. The Unfolded Protein Response

**Figure 8:**
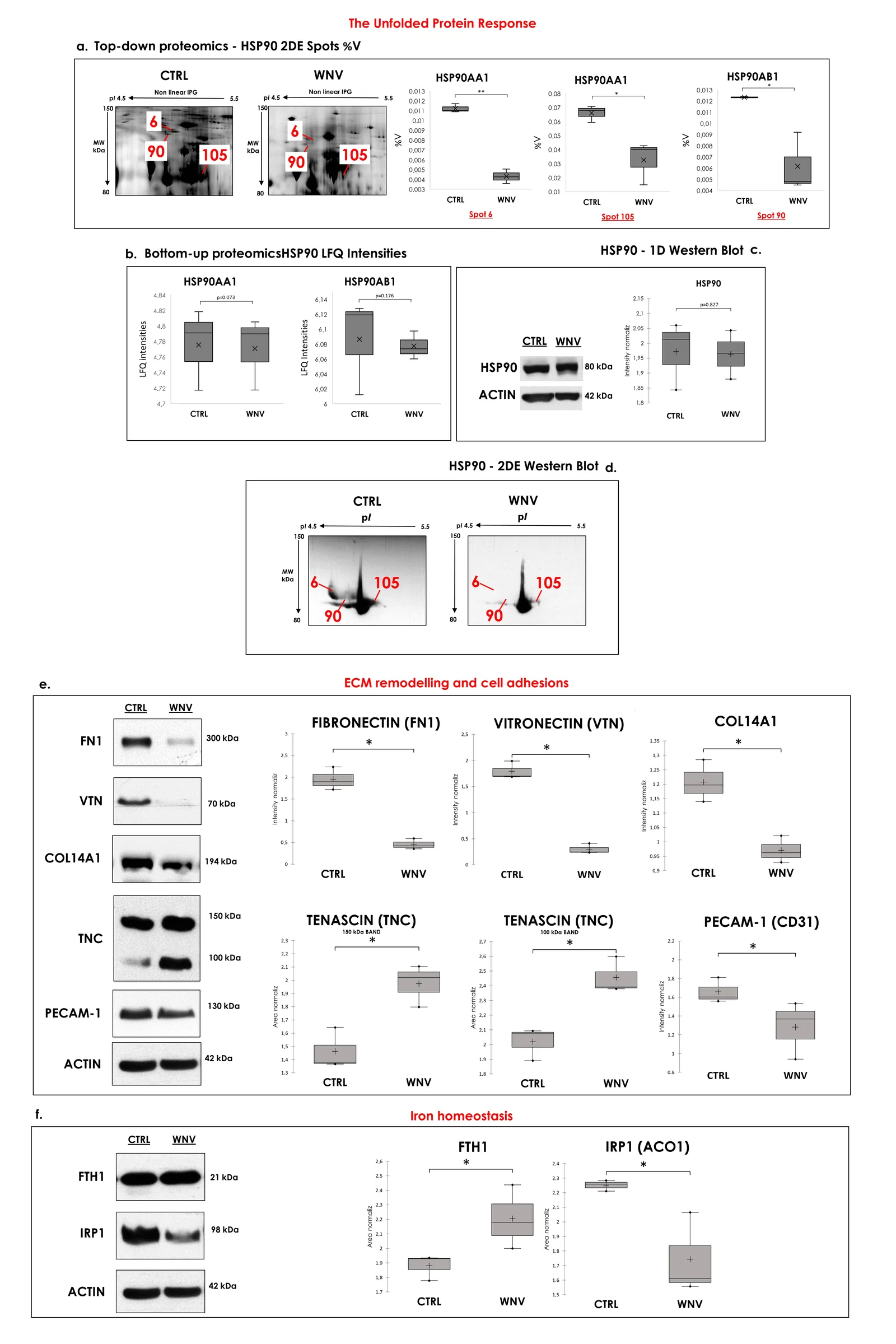
Western blot validations. (**a**) HSP90 (HSP90AA1, alpha; HSP90AB1, beta) protein species are shown in representative 2DE CTRL and WNV gels (spot numbers are marked in red and correspond to spot numbers in Table S2) on the left; box plots of relative %V for each spot in both CTRL and WNV conditions. ANOVA test was performed with *p*-value ≤ 0.05 (*) or ≤ 0.01 (**), on the right. (**b**) Box plots of HSP90 alpha and beta as total protein using LFQ intensities in CTRL and WNV samples by label-free quantitative analysis. ANOVA test was performed, but no significant variation was observed. (**c**) Western blot (WB) of HSP90 in CTRL and WNV samples. Normalization on actin as housekeeping. On the right, box plot of normalized intensities. ANOVA test was performed, but no significant variation was observed. (**d**) Two-dimensional WB of HSP90 protein species in CTRL and WNV samples. (**e**) ECM remodeling and cell adhesion regulation in WNV infection. Proteins tested in CTRL and WNV samples: fibronectin (FN1), vitronectin (VTN), collagen alpha-1(XIV) chain (COL14A1), tenascin (TNC), and PECAM-1 (platelet endothelial cell adhesion molecule). (**f**) The iron homeostasis modulation in WNV infection. Proteins tested in CTRL and WNV samples: ferritin heavy chain (FTH1) and cytoplasmic aconitate hydratase (IRP1/ACO1). Normalization on actin as housekeeping. On the right, box plots of normalized signals. ANOVA test was performed, and significant *p*-value ≤ 0.05 is marked with *.

One relevant modulated molecular pathway is protein folding and the UPR, as especially suggested by the top-down results: for example, a significant variation in the abundance of protein species of HSP90 (spot 6 and spot 105 for HSP90AB1; spot 90 for HSP90AB1), HSPA8 (spot 65), HSPD1 (spot 1), HSPA14 (spot 15), HYOU1 (spots 11, 18 and 28), and VCP (spot 114), as reported in Table S2. All these proteins were properly identified by LC-MS/MS in the bottom-up analysis; however, no quantitative differences were confirmed with a fold change ≥ 1.8. Given these results, further experiments were performed to validate the dysregulation of the UPR, focusing on HSP90. Through the top-down approach, three protein species of HSP90 (subunits alpha and beta) were observed to be significantly downregulated after WNV infection (Figure 8a), which suggests that HSP90 is subjected to differential post-translational modulation resulting from WNV infection. On the other hand, through the bottom-up approach, no significant variation in the LFQ intensities of HSP90AA1 and HSP90AB1 at the total protein level was reported, despite a slight decrease in the intensity of HSP90AB1 (Figure 8b). These results were also confirmed by 1D-WB anti-HSP90 of different biological replicates (Figure 8c). Given these data, HSP90 protein species were investigated by 2D-WB, which allowed us to observe protein species with different PTMs as they differ for p*I* and MW. As shown in Figure 2 and Figure 8 dD-WB validated the decrease in abundance of some specific protein species of HSP90 after WNV infection (spots 6, 90 and 105; Table S2).

#### 2.5.2. ECM Remodeling and Cell Adhesions

Furthermore, enrichment analyses of the bottom-up proteomic data highlighted the dysregulation of other molecular mechanisms as a consequence of WNV infection and replication: ECM remodeling and cell–cell adhesions. These results were further validated by 1D-WB (Figure 8e), which specifically targeted some differential proteins involved in the pathways of interest, as well as other proteins involved in the target mechanisms, to obtain more robust and comprehensive results. Among the differential proteins associated with ECM remodeling and cell–matrix interaction, fibronectin 1 (FN1) and vitronectin (VTN) were confirmed by 1D-WB to be significantly downregulated after WNV infection. Moreover, a robust and significant decrease in the abundance of COL2A1 was confirmed by both the top-down and bottom-up approaches (Figure 7a), as reported previously. Additionally, we tested collagen alpha-1(XIV) chain (COL14A1), taken into consideration due to its role as a fine regulator of ECM architecture [17], and a significant decrease was confirmed by 1D-WB after viral infection (Figure 8e). Another ECM protein considered was tenascin-C (TNC), investigated by WB, because it is known to be rapidly upregulated in response to infection, tissue injury, and neuro-inflammation, where it functions as a key modulator of innate immune responses and tissue remodeling [18,19]. Through 1D-WB, no detectable band at the proper MW (240 kDa) was observed in either the uninfected or infected samples; however, two bands at 150 and 100 kDa (hypothetically, bioactive proteolytic isoforms) were detected, and both were observed to be significantly upregulated in WNV-infected samples (Figure 8e). In support of this evidence of ECM remodeling, an increased abundance of arylsulfatase B (ARSB), involved in chondroitin-4-sulfate (C4S) degradation, was also reported by the bottom-up proteomic approach after viral infection (Table S3). Moreover, the bottom-up results highlighted the decreased abundance of several proteins involved in desmosome and intermediate cell junctions, such as plakophilin-1 (PKP1), junction plakoglobin (JUP), desmocollin-1 (DSC1) and desmoplakin (DSP), as well as the dysregulation of tight junctional proteins, such as the decreased level of junctional adhesion molecule A (F11R/JAM1) and the increased level of epithelial cell adhesion molecule (EPCAM) (Table S3). As a further validation of these results, an immunoblotting against platelet endothelial cell adhesion molecule (PECAM1) was performed, as PECAM1 is a cell adhesion molecule with a key role in leukocyte trans-endothelial migration [20] and contributes to changes in barrier integrity during inflammatory conditions [21,22]. WB analysis reported a significant decrease in the abundance of PECAM1 in WNV-infected samples (Figure 8e).

#### 2.5.3. Iron Homeostasis

Interestingly, enrichment analyses of the bottom-up differential proteins highlighted the dysregulation of iron transport, as suggested by the increased abundance of ferritin heavy chain (FTH) and the decreased abundance of iron-responsive element-binding protein 2 (IREB2/IRP2), as reported in Table S3. FTH1, IREB2 and ACO1/IRP1 are part of the iron regulatory protein (IRP)—iron responsive element (IRE)—system, as FTH1 exhibits its main function in the storage of excessive intracellular iron in a soluble and non-toxic form. IREB2 and IRP1 are RNA-binding proteins that bind iron-responsive elements (IRES) of ferritin and transferrin receptor mRNAs, regulating their translation according to intracellular iron levels [23]. As a validation of this pathway, we performed WB analysis of FTH1 and IRP1, and the results confirmed the statistically significant increase and decrease in abundance, respectively, after viral infection (Figure 8f).

## 3. Discussion

Proteomics has been gaining increasing interest in viral studies, given its powerful ability to elucidate various aspects of viral infections, starting from virus–host interactions, viral replication mechanisms, the cellular response to the infection, viral strategies of evading the immune system, and antiviral drug target identification and diagnostics. Integrating bottom-up and top-down approaches provides complementary information: individually, bottom-up might provide a large-scale characterization of the host proteome, while top-down enables the characterization of protein species, focusing on PTMs; therefore, an extensive proteomic strategy composed of the combination of the two approaches might provide a more comprehensive overview of the molecular processes regulating and opposing viral infection [11]. In the context of flaviviruses, and particularly WNV, proteomics has increasingly been used to investigate not only proteomic perturbations induced by the virus and the molecular mechanisms underlying this infective process but also the specific protein modifications involved in the viral cycle. With this purpose, other studies have been carried out previously on non-human cells, such as rat, mouse and monkey cells, by the application of 2D-DIGE technology and MS as top-down proteomic approach [24,25,26]. Interestingly, these studies identified different protein species of WNV proteins, supporting the ability of a top-down methodology in distinguishing and identifying viral proteins with different PTMs. This evidence is in line with our results, as we identified four protein species of NS3 (spots 23, 53, 57 and 70) and four protein species of NS1 (spots 3, 5, 7 and 69). Indeed, PTMs on viral proteins regulate several aspects of the WNV lifecycle and pathogenesis [27], as demonstrated by multiple studies: glycosylation of envelope protein [28,29] or of NS1 [30] contributes to viral pathogenesis; phosphorylation or ubiquitination of capsid protein [31,32] regulates capsid–RNA binding or its degradation; acetylation of NS3 regulates its helicase activity [33]; and phosphorylation of NS5 modulates viral RNA replication [34].

Extensive enrichment analysis of our proteomic data sets highlighted the significant regulation of translation initiation following WNV infection, associated with a relevant dysregulation of protein folding processes and, especially, the UPR. With regard to translation initiation processes, PPP1CC/PP1γ was found to be significantly decreased in abundance after infection in both the top-down and bottom-up analyses (Figure 7a, Tables S2 and S3), although the top-down proteomic results provided additional supporting data, such as the decreased abundance of eukaryotic translation initiation factors as protein species of EIF1, EIF3F, EIF3G, and EIF3K and the increased abundance of protein species of EIF3E and EIF4A1. PPP1CC is a phosphatase that dephosphorylates EIF2α, promoting translation initiation. Indeed, the decreased level of PPP1CC could be included in an overall antiviral response in order to inhibit translation, therefore interfering with the viral lifecycle [35]. On the other hand, its decreased abundance might also reflect an immune evasion strategy mediated by WNV itself. In fact, other studies reported that some RNA viruses, such as the measles virus and WNV as well, escape the innate immune defense by targeting the phosphatases PP1α and PP1γ, as they mediate the dephosphorylation of the cytosolic sensor MDA5, promoting their activity and the transcription of IFN α/β [36,37]. Furthermore, the observed decreased abundance of several members of the eIF3 complex and EIF1 might suggest a viral strategy established to partially “shut off” the translation of host proteins, hijacking cellular translation machineries to favor viral RNA and protein synthesis or blocking the translation of antiviral response proteins [25]. In fact, the virus still needs cellular machineries to support its protein synthesis, and this might be supported by an increased abundance of EIF4A1, a crucial member of the eIF4F complex, known to be engaged by the virus to support its protein synthesis [38].

In this scenario, the massive production of viral proteins in support of the formation of new virions might lead to an overloaded ER, triggering the activation of the ER-associated protein degradation (ERAD) pathway and the UPR [39,40]. Among the differential proteins involved with this pathway, the top-down approach highlighted a dysregulation of cellular chaperones after WNV infection, such as some protein species of HSP90, HSPA8, HSP60 and HSP70. Interestingly, HSP70 has already been described as a negative regulator of WNV capsid protein via direct interaction [41], which supports our evidence as a potential cytoprotective cellular mechanism against the virus. However, the decreased abundance of HSP90 protein species, as demonstrated by our proteomic and validation results and also reported in WNV-infected VERO cells [25], might be another viral strategy to evade the innate immune response. Indeed, as reported by Roby et al., flaviviruses, including WNV, use viral NS5 to dysregulate HSP90-JAK interactions, antagonizing JAK/STAT signaling downstream of IFN α/β [42].

As a small proportion of infections progresses to severe neuroinvasive disease, characterized by encephalitis and meningitis, several strategies have been suggested to explain WNV neuroinvasion [5,43]. One theory involves the BBB disruption and increased permeability induced by WNV infection via the dysregulation of tight junction proteins of brain endothelial cells [44]. According to our results, especially the experimental bottom-up proteomic data, WNV-infected cells present a massive decrease in the abundance of cell adhesion molecules, for instance, JAM1 as a tight junction protein and tyrosine-protein kinase Yes (YES1) as a regulator of tight junctions’ assembly; other downregulated junctional proteins include members of desmosomes, such as JUP, PKP1, DSC1 and DSP. These findings are consistent with previously reported alterations in cell junction-associated proteins during WNV infection, including observations in the brains of WNV-infected mice [45], although their direct relevance to blood–brain barrier dysfunction remains to be established. Although it is still debated whether increased BBB permeability may be a consequence or a cause of the virus’s entry into the CNS, several studies demonstrated, in WNV infection models, a significantly increased expression and production of matrix metalloproteinases (MMPs), which might mediate the degradation of the ECM components of the BBB and contribute to viral dissemination and neuroinvasion [43,46]. For instance, Verma et al. reported a significantly increased mRNA expression of MMP-1, -3 and -9 in WNV-infected human brain cortical astrocytes, especially between 2 and 4 days after infection, suggesting that MMP expression is influenced also by viral replication in addition to viral entry itself [47]. In support of this experimental evidence, increased MMP9 expression and enzymatic activity was reported also in brains of WNV-infected mice, as well as in cerebrospinal fluid from patients with WNV infection [48]; moreover, similar results were also reported for other flaviviruses, such as ZIKV [49], DENV [50] and JEV [51]. Interestingly, our proteomic results, together with WB validation, highlighted the significant decrease in WNV-infected Huh7 cells of collagen protein COL2A1 and of COL14A1 as an ECM architecture regulator, as well as FN1 and VTN, suggesting that WNV infection might induce ECM remodeling also by reducing the intracellular production of the ECM components themselves. An alteration in collagen metabolism at the protein level was also reported by Besson et al. in human neuronal models infected with either WNV or JEV [52]. Moreover, FN1 and VTN are glycoproteins playing physiopathological roles in the ECM, modulating cell adhesion, cell migration and immune responses. Because multiple studies reported their involvement in virus interactions with host cells, or their role in suppressing or evading the immune response [53,54,55], our evidence of the modulation of these proteins at the protein level after WNV infection warrants further investigation.

Another hypothesis regarding the basis of WNV neuroinvasion relies on the potential role of infected immune cells, especially monocytes and macrophages, as “Trojan horse” cells, as they adhere to and migrate across the disrupted or highly permeable BBB, favored by the increased expression of adhesion molecules, such as VCAM-1, and reduced cell-to-cell interactions [43,56,57]. This idea might, indeed, be supported by our results, which specifically report a decreased abundance of PECAM-1 and an increased abundance of EPCAM at the protein level in WNV-infected cells.

Another interesting aspect arising from recent scientific research in host–pathogen studies is the so-called “nutritional immunity”, which refers to a particular host response characterized by the inhibition of pathogen access to key factors, such as iron. Indeed, iron has a crucial role in several host and viral metabolic processes [58]. In viral infections, excess iron might be used to promote viral replication and enhance its pathogenicity, while host cells may modify the iron availability to interfere with the viral lifecycle [59]. Our analysis indeed highlighted a modulation of intracellular iron homeostasis after WNV infection: on the one hand, a significant increase in the abundance of ferritin heavy chain 1 (FTH1) in order to store the excess of intracellular iron and prevent oxidative damage; on the other hand, a significant decrease in the abundance of the iron-responsive element-binding protein 2 (IREB2/IRP2) and cytoplasmic aconitate hydratase (IRP1/ACO1) in order to allow the translation of proteins involved with iron metabolism. Interestingly, Duchemin et al. demonstrated that WNV-infected *Culex* mosquitoes’ cells respond to infection through an increased expression of FTH1, suggesting its role in sequestering intracellular iron and therefore reducing its availability to WNV as a host defense mechanism [60].

**Figure 9:**
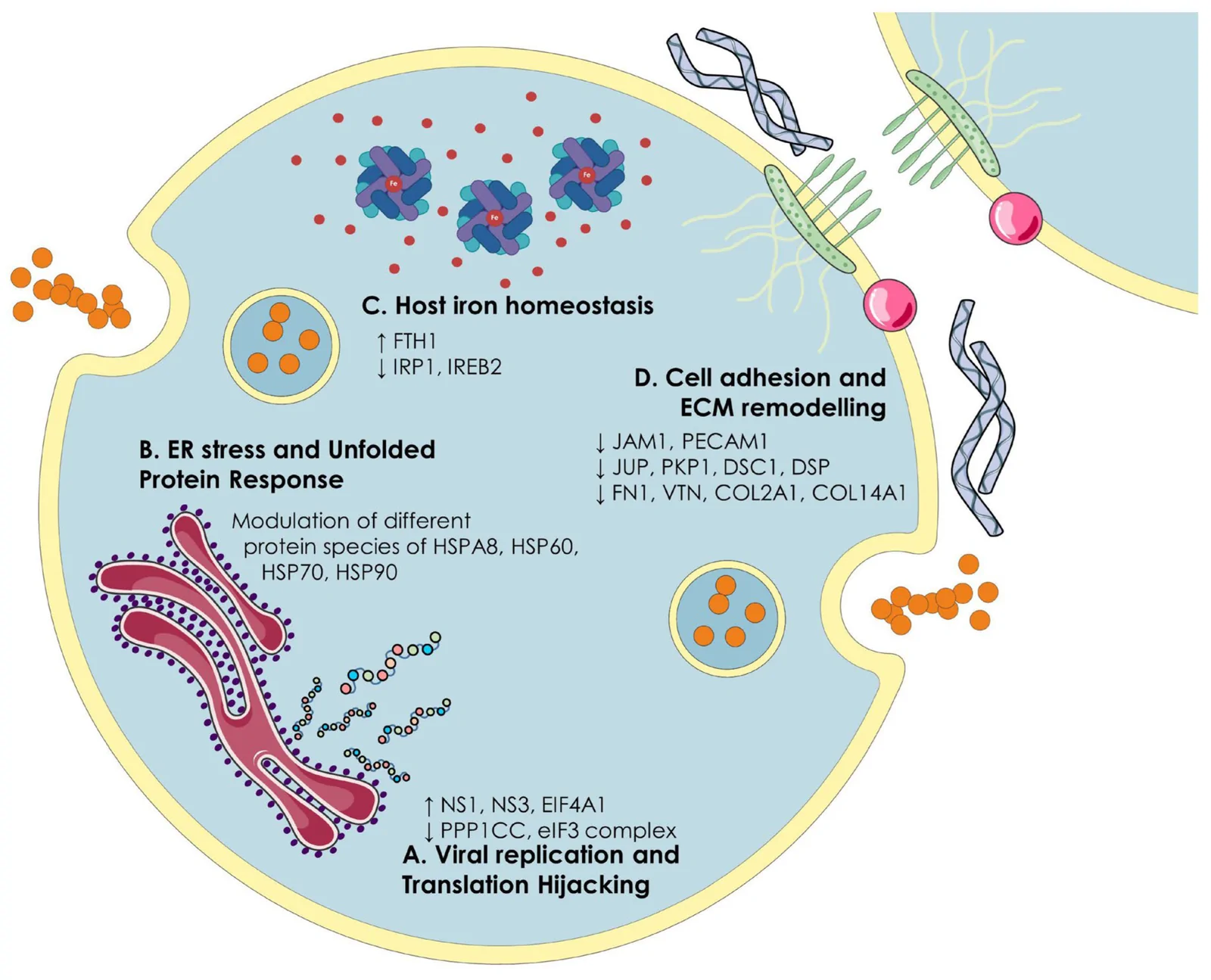
Schematic model of host modulation pathways upon WNV infection and their contribution to viral pathogenesis. (**a**) Upon infection, WNV hijacks host translation machinery (modulation of eIF3 complex, PPP1CC, and EIF4A1) to support its viral replication and protein synthesis (increase in viral NS1 and NS3); (**b**) protein overload in the ER triggers the UPR (dysregulation of HSPA8, HSPD1, HSP70, and HSP90); (**c**) host cells modulate iron homeostatic processes (increase in FTH1, decrease in IRP1 and IREB2) to sequester free iron, essential for optimal viral replication; and (**d**) widespread decrease in cell–cell junctional proteins (JAM1, PECAM1, and desmosomes) and ECM components (FN1, VTN, and COL2A1/14A1) leads to the breakdown of the cell structure’s integrity.

Although the use of Huh7 cells represents a limitation in terms of extrapolating our findings to WNV neuropathogenesis, their selection was supported by our previous experience demonstrating their high permissiveness to WNV infection [61], as well as by previous proteomic studies employing Huh7 cells to investigate flavivirus–host interactions [62]. Moreover, proteomic investigations of WNV infection have also been performed in non-neuronal cell models [25]. Therefore, Huh7 cells were considered an appropriate experimental model for investigating WNV-induced proteomic alterations. Nevertheless, the observed changes, particularly those involving cell adhesion and extracellular matrix proteins, cannot be directly extrapolated to mechanisms of blood–brain barrier disruption or neuroinvasion. Further studies in neuronal and brain endothelial cell models would be necessary to establish their relevance to WNV neuropathogenesis. Given these considerations, we could summarize our evidence in a schematic host cellular context in which proteomic alterations could reflect WNV pathogenic processes upon cell infection. As shown in Figure 9, WNV infection impacts cellular processes either to support or to counteract viral processes: the dysregulation of proteins of the host translation machinery (decrease in eIF3 complex and PPP1CC, increase in EIF4A1, and increase in viral NS1 and NS3) supports the hypothesis of the viral hijacking of host translation to optimize viral polyprotein synthesis while reducing host antiviral proteins. As a consequence, viral protein overload might lead to ER stress and to the UPR (dysregulation of HSPA8, HSP60, HSP70, and HSP90). Dysregulation of iron homeostasis proteins (increase in FTH1, decrease in IRP1 and IREB2) might represent a host strategy to sequester free cytosolic iron, depriving the virus of essential metallic cofactors for replication. Moreover, an overall decrease in junctional proteins, such as desmosomal components (JUP, PKP1, DSC1, and DSP) and tight junctional proteins (JAM-1, PECAM1), as well as ECM matrix components (FN1, VTN, COL2A1, and COL14A1), represents a conserved mechanism of impairment of the cell structure’s integrity.

Notably, the application of an extensive proteomic approach provided a deeper overview of the molecular mechanisms carried out by host cells to counteract the infection, as well as the processes exploited by the WNV to survive. Importantly, the integration of both top-down and bottom-up proteomic strategies offered complementary insights, enabling a more comprehensive characterization of protein species, PTMs and overall protein dynamics.

## 4. Materials and Methods

### 4.1. Experimental Design

The extensive proteomic approach was carried out by experiments designed to reduce experimental variability and ensure a robust comparison between top-down and bottom-up proteomic data. In detail, three independent (*n* = 3) Huh7 cell cultures for each condition, control and WNV-infected, were prepared, and each biological replicate was split equally into two aliquots, one subjected to top-down proteomic analysis and the other one to bottom-up analysis. Top-down proteomic analysis was carried out using three biological replicates per condition to avoid experimental variability due to batch effect. Bottom-up proteomic analysis was carried out using three biological replicates per condition, each of which had been run by LC-MS/MS in three technical replicates (*n* = 3) to avoid potential technical errors. Statistical testing was then performed on independent biological triplicates per condition, and statistical significance was set at *p*-value ≤ 0.05 for differential proteins. Further statistical parameters applied to each approach are provided below. Furthermore, different biological replicates (*n* = 3) per condition were used for Western blot validations.

### 4.2. Cell Culture and WNV Infection

Huh7 cells (Istituto Toscano Tumori, Core Research Laboratory, Siena, Italy) were used to assess WNV infection. A total of 3 × 10^5^ cells were pre-seeded in 6-well culture plates and maintained in Dulbecco’s modified Eagle’s Medium (DMEM, Euroclone, Milan, Italy) supplemented with 10% fetal bovine serum (FBS, Euroclone) and 1% of penicillin/streptomycin (Pen/Strep, Euroclone). Specifically, three independent cell cultures per condition (CTRL and WNV-infected) were prepared, intended for both top-down and bottom-up proteomic analyses. Concerning WNV infection, WNV lineage 1 strain Italy/2009 was adsorbed at 0.005 MOI on pre-seeded Huh7 cells for 1 h at 37 °C with 5% CO_2_. Then, the viral stock was removed and replaced with infection medium (DMEM supplemented with 1% FBS and 1% Pen/Strep) and incubated for 48 h at 37 °C with 5% CO_2_. After the incubation, CTRL and infected cells were harvested by trypsinization. Cells were centrifuged at 300× *g* for 5 min at room temperature and washed with 1× PBS, and the procedure was repeated three times. After the final washing, cell pellets were inactivated at 95 °C for 5 min and stored at −80 °C until proteomic analysis. The infection conditions (MOI 0.005 and 48 h post-infection) were selected based on preliminary experiments evaluating cell seeding density, cytopathic effects, and viral replication (Supplementary Table S1). It is important to note that the CTRL group was subjected to the same experimental procedures as the WNV-infected group, including the adsorption step, medium changes, and subsequent sample processing and inactivation procedures, with the only difference being the absence of virus. Therefore, the CTRL group represents a true mock-infected control.

### 4.3. Top-Down Proteomic Analysis

#### 4.3.1. Two-Dimensional Electrophoresis

For top-down proteomic analysis by 2DE, samples were prepared using a lysis buffer composed of 7 M urea, 2 M thiourea, 4% 3-[(3-cholamidopropyl) dimethylammonia]-1-propanesulfonate hydrate (CHAPS) (*w*/*v*), 1% (*w*/*v*) dithioerythritol (DTE) and 0.1% (*v*/*v*) Triton X-100. Protein concentration was assessed by Bradford assay. 2DE was performed as previously described in Gagliardi et al. [63].

#### 4.3.2. Image and Statistical Analyses

Image analysis of the 2DE gels was performed using Melanie™ Classic 9 (SIB Swiss Institute of Bioinformatics, Geneva, Switzerland). Experimental design includes the comparison between control healthy cells and WNV-infected cells. Therefore, differential statistical analysis was carried out by one-way ANOVA test with Benjamini–Hochberg FDR-adjusted post hoc tests using XLStat (Addinsoft, Paris, France, version 2021.5.1). Statistical testing was performed taking into account the percentage of relative volume (%V) of each spot and a *p*-value ≤ 0.05 and a fold change ≥ 1.8. PCA by Pearson’s correlation and heatmap analysis by Euclidean distance were carried out using %V of differential protein species by XLStat.

#### 4.3.3. Protein Identification by MALDI-ToF MS

Differential protein species were manually excised from MS-preparative 2DE gels of both control and WNV-infected cells in order to increase robustness of protein identification, especially of viral protein species. Protein spot preparation and MALDI-ToF mass spectra acquisition were carried out as previously described by Shaba et al. [64]. The obtained mass spectra were analyzed by FlexAnalysis software version 3.0 (Bruker, Brema, Germany), and peak lists were created according to the following parameters: Snap as peak detection algorithm, smoothing by SavitzkyGolay algorithm (0.2 *m*/*z* width, 1 cycles), and signal-to-noise threshold set at 6. Peak lists were, first, internally calibrated on “Trypsin_Promega_porcine mass control list”, then filtered out of mass lists for contaminants, such as matrix-related ions, keratin peaks and tryptic peaks. Protein identification was carried out by PMF through Mascot Search Engine (Matrix Science Ltd., London, UK, http://www.matrixscience.com; accessed on 5 September 2024), using the following parameters: Homo sapiens or Other Viruses as taxonomy, SwissProt as database, trypsin as cleavage enzyme, 20 ppm as mass tolerance, one admissible missed cleavage site, carbamidomethylation (iodoacetamide alkylation) of cysteine as fixed modification and oxidation of methionine as variable modification. *p*-value ≤ 0.05 (reported as “expect” in Table S2) and score ≥ 55 were used as minimum criteria for protein identifications. The mass spectrometry proteomics data have been deposited into the ProteomeXchange Consortium (http://proteomecentral.proteomexchange.org) via the PRIDE partner repository [65] with the data set identifier PXD080239.

### 4.4. Bottom-Up Proteomic Analysis

#### 4.4.1. Sample Preparation for LC-MS/MS

Cellular pellets were suspended in lysis buffer containing 5% (*w*/*v*) SDS and 50 mM triethylammonium bicarbonate (TEAB) and then sonicated for 10 s on ice using ultrasonic processor with 70% amplitude (Model 120 Sonic Dismembrator, Fisherbrand, Thermo Fisher Scientific, Waltham, MA, USA), twice. Samples were centrifuged at 13,000× *g* for 8 min at RT, and protein concentration was determined using a BCA assay kit according to manufacturer’s protocol. For each biological replicate, 50 µg of protein was subjected to LC-MS/MS preparation by S-Trap technology (ProtiFi, Fairport, NY, USA). First, samples were reduced with 120 mM tris(2-carboxyethyl)phosphine (TCEP) for 15 min at 55 °C, alkylated with 500 mM methyl methanethiosulfonate (MMTS) (alkylating solution in LC-MS grade isopropanol) for 10 min at RT, and, lastly, acidified with 27.5% (*v*/*v*) phosphoric acid. Successively, binding/wash buffer (composed of 100 mM TEAB in 90% LC-MS-grade methanol) was added to samples, and the whole solution was transferred into the S-trap micro spin column, followed by a brief centrifugation at 4000× *g* at RT for 30 s to trap proteins. Three washes with binding/wash buffer were performed, each wash followed by a centrifugation at 10,000× *g* at RT for 30 s; lastly, an additional centrifugation at 10,000× *g* at RT for 1 min was performed to fully remove the resting buffer. At this point, samples were digested overnight with trypsin at a ratio of 1:10 (protease-to-protein ratio) at 37 °C. Subsequently, peptides were eluted from columns as follows: first, a solution composed of 50 mM TEAB was added and columns were centrifuged at 10,000× *g* at RT for 1 min; then, a solution composed of 50% (*v*/*v*) acetonitrile and 50 mM TEAB was added, followed by a centrifugation at 10,000× *g* at RT for 1 min. The last step was then repeated. Eventually, eluted peptides were concentrated and dried in SpeedVac and frozen at −80 °C until LC-MS/MS run.

#### 4.4.2. Label-Free Quantitative Proteomic LC-MS/MS Analysis

Each peptide mixture was resuspended in 200 µL of 0.2% HCOOH, and 1 µL was analyzed by LC-MS/MS using a last-generation mass spectrometer equipped with Orbitrap analyzer EXPLORIS 240 (ThermoScientific, Waltham, MA, USA) coupled to a Vanquish-Neo UHPLC system. After loading, the peptide mixture was first concentrated and desalted on the pre-column (PepMap™ Neo Trap Cartridge). Each peptide sample was fractioned on a C18 reverse-phase capillary column (Double nanoViper™ PepMap™ Neo 2 µm C18 75 µm × 150 mm) working at a flow rate of 250 nl/min in a gradient from 5% to 95% of eluent solvent B (eluent B: 0.2% formic acid in 95% acetonitrile; eluent A: 0.2% formic acid and 2% acetonitrile in ultrapure water) over 77 min. The MS/MS acquisition method was set up in a Data-Dependent Acquisition mode, with a full scan ranging from 300 to 1800 *m*/*z*. Three technical replicates for each of the three biological replicates per condition (uninfected and infected) were run.

#### 4.4.3. Protein Identification and Quantification

Raw files were analyzed by MaxQuant software (version 2.6.5.0), and the obtained mass spectra were searched with the built-in Andromeda search engine against two proteomes: *Homo sapiens* reference proteome (UPS000005640, downloaded in October 2025; 83,516 sequences) and West Nile Virus proteome (UPS000096925, downloaded in October 2025; 1 sequence) from Uniprot (https://www.uniprot.org). Methionine oxidation was set as variable modification, while beta-methylthiolation on cysteine was specified as fixed modification; moreover, a maximum number of 5 modifications per peptide was allowed. Trypsin/P was specified as the cleavage enzyme, with a maximum number of two missed cleavages allowed. Protein identification included the following minimum criteria: main search peptide tolerance 4.5 ppm for precursor ions and mass tolerance 20 ppm for fragment ions, a minimum peptide length of 5 amino acids, a maximum peptide mass of 4600 Da and a minimum number of two unique peptides. “Match between runs” mode was enabled with a match time window of 0.4 min and an alignment time window of 20 min. A false discovery rate (FDR) was adjusted to 0.01, both at peptide and protein levels, in combination with target-decoy mode (revert). A relative label-free quantification of proteins was carried out by the MaxLFQ algorithm integrated in the software using a LFQ minimum ratio count of two. The MS proteomics data have been deposited into the ProteomeXchange Consortium (http://proteomecentral.proteomexchange.org) via the PRIDE partner repository [65] with the data set identifier PXD080593.

#### 4.4.4. Statistical Analysis

The analysis of MaxQuant-processed data was carried out by Perseus (version 2.1.3.0) using “proteingroups.txt” file. Protein identifications marked as reverse sequences, contaminants and proteins only identified with modified peptides were removed from the matrix. Two categorical annotations were introduced, grouping samples according to belonging biological replicate and then, also, to belonging condition (uninfected, CTRL; infected, WNV). LFQ intensities were transformed to a log scale (Log_2_) and then normalized across samples by subtraction of median. Only those proteins presenting valid values at 70% in at least one condition, CTRL or WNV, were used for further statistical processing. Missing values were imputed from a normal distribution (width of 0.3 and down-shift of 1.8 in standard deviations). Differential analysis between CTRL and WNV biological triplicates was carried out by Student’s *t*-test (S0 0.1; FDR 0.05) with multiple hypothesis testing correction using permutation-based FDR. Furthermore, among statistically significant (*p*-value ≤ 0.05) differential proteins, only those with a fold change ≥ 1.8 were considered for bioinformatic analyses. Multivariate statistical analysis was carried out: histograms of LFQ values, PCA and multi-scatterplot analysis by Pearson’s correlation were performed by Perseus software, while heatmap analysis by Euclidean distance was performed by XLStat (Addinsoft, Paris, France).

#### 4.4.5. Bioinformatic Analyses

Bioinformatic analyses of top-down and bottom-up differential proteins, independently and in comparison, were performed by MetaCore software (https://portal.genego.com, accessed on 25 October 2025) (Clarivate analytics, Boston, MA, USA) and EnrichR tool (https://maayanlab.cloud/Enrichr, accessed on 25 October 2025) by submitting the UniProt accession number (AN). Enrichment analysis of top-down differential proteomic data set was performed by pathway maps, process networks, GO biological processes and GO molecular function; enrichment analysis of bottom-up proteomic data set was performed by pathway maps and process networks, GO biological processes and GO cellular compartment. Both differential proteomic data sets were analyzed to recover potential in silico interactors to WNV viral proteins by virus–host interaction PPI P-HIPSTer algorithm by submitting gene names to EnrichR tool: this computational algorithm predicts virus–host protein–protein interactions (PPIs) using sequence and structure-based information [66].

#### 4.4.6. Western Blot Validations

As further step, validations of specific differential proteins and of proteins related to specific pathways modulated after WNV infection were performed by Western blot. Aliquots of thirty µg of proteins extracted from different CTRL and WNV-infected cells, assessed in biological triplicate, were used. Samples were dissolved in 20 µL of LAEMMLI (Tris HCl pH 6.8 62.5 mM, 20% *v*/*v* glycerol, 2% *w*/*v* SDS, 5% *v*/*v* β-mercaptoethanol, 0.032% *w*/*v* bromophenol blue) then kept at 95 °C for 7 min and, eventually, loaded in 10% (made in-house) or 4–15% gradient polyacrylamide gels (Bio-Rad Laboratories, Hercules, CA, USA). SDS-PAGE was carried out using the Mini-PROTEAN electrophoresis system (Bio-Rad). After electrophoresis, gels were equilibrated in transfer buffer (Tris 25 mM, glycine 192 mM, 20% *v*/*v* methanol) for 1 h and transferred to nitrocellulose membranes (Cytiva, MA, USA). In order to assess transfer and equal protein loading, membranes were stained with Red Ponceau (0.2% *w*/*v* Ponceau S in 3% *v*/*v* acetic acid). 2D Western blots were performed as described by Ponzone L. et al. [67], using one biological replicate per condition, CTRL and WNV. Membranes were tested with different primary antibodies: anti-HSP90 (mouse monoclonal; 60318-1-Ig; Proteintech, Rosemont, IL, USA; dilution 1:5000); anti-fibronectin/FN1 (rabbit monoclonal; 26836; Cell Signaling, Danvers, MA, USA; dilution 1:1000); anti-vitronectin (rabbit monoclonal; 82821; Cell Signaling; dilution 1:1000); anti-PECAM-1/CD31 (mouse monoclonal; 3528; Cell Signaling; dilution 1:1000); anti-tenascin C (rabbit monoclonal; 33352; Cell Signaling: dilution 1:1000); anti-COL14A1 (rabbit monoclonal; 61964; Cell Signaling; dilution 1:1000); anti-FTH1 (rabbit monoclonal; 4393; Cell Signaling; dilution 1:1000); and anti-IRP1/ACO1 (rabbit monoclonal; 20272; Cell Signaling; dilution 1:1000). Specifically, membranes were analyzed with different secondary antibodies, such as IgG peroxidase-conjugated goat anti-mouse at 1:30,000 dilution (A2554, Sigma-Aldrich, Saint Louis, MO, USA) and IgG peroxidase-conjugated goat anti-rabbit at 7000 dilution (A4914, Sigma-Aldrich). Normalization to housekeeping protein was performed by immunoblotting anti-β-actin (mouse monoclonal; A5441, Sigma-Aldrich; dilution: 1:40,000). ECL chemiluminescence detection system (Cytiva) was utilized to detect specific immunoreactive proteins, and images were analyzed with ImageJ (1.51p, NIH, Chicago, IL, USA) or Melanie™ Classic 9. Statistical analysis was performed by XLSTAT (Addinsoft).

## 5. Conclusions

Given the absence of effective antiviral strategies, in association with the rapid worldwide diffusion of WNV, a more profound understanding of WNV pathogenic activities in human host cells is crucial. To this purpose, this study provides the first extensive proteomic characterization of WNV infection in human cells by integrating top-down and bottom-up proteomic approaches. This combined approach revealed that WNV induces profound host cellular alterations, ranging from the large-scale modulation of the host proteome to specific PTMs of viral and cellular proteins. Our key findings highlight the virus’s ability to hijack host machinery to its advantage, which triggers the UPR and a dysregulation of translation initiation. Notably, top-down analysis itself revealed that the modulation of this biological process is finely regulated by modifications at the post-translational level. Furthermore, the observed downregulation of junctional proteins and ECM components, as highlighted by the bottom-up approach, provides molecular evidence for viral invasion and viral neuroinvasive strategies. In addition, we also provided evidence of a modulation of host iron homeostasis, as a host defense mechanism potentially related to the inhibition of pathogen access to key factors such as iron, which compromises the viral lifecyle. This evidence might pave the way for further investigations to explore the potentiality of ”nutritional immunity”. In conclusion, these results offer valuable insights into WNV pathogenesis and shed light on potential antiviral targets.

## Data Availability

All mass spectrometry proteomics data have been deposited into the ProteomeXchange Consortium via the PRIDE partner repository, with the data set identifiers PXD080239 for the top-down data set and PXD080593 for the bottom-up data set. All other relevant data are within the paper and its Supporting Information files.

## Supplementary Materials

The following supporting information can be downloaded at: https://www.mdpi.com/article/10.3390/ijms27209387/s1.
