# Supplementary material for: Extensive Proteomic Approach to Assess Protein Alterations in Human Hepatoma Huh7 Cells Infected with West Nile Virus
Source: Int J Mol Sci. 2026 Oct 21;27(20):9387. doi: 10.3390/ijms27209387 (PMC13705241; doi:10.3390/ijms27209387)
Supplement: Supplementary file 1 [file ijms-27-09387-s001.zip › Supplementary Figures.pdf]

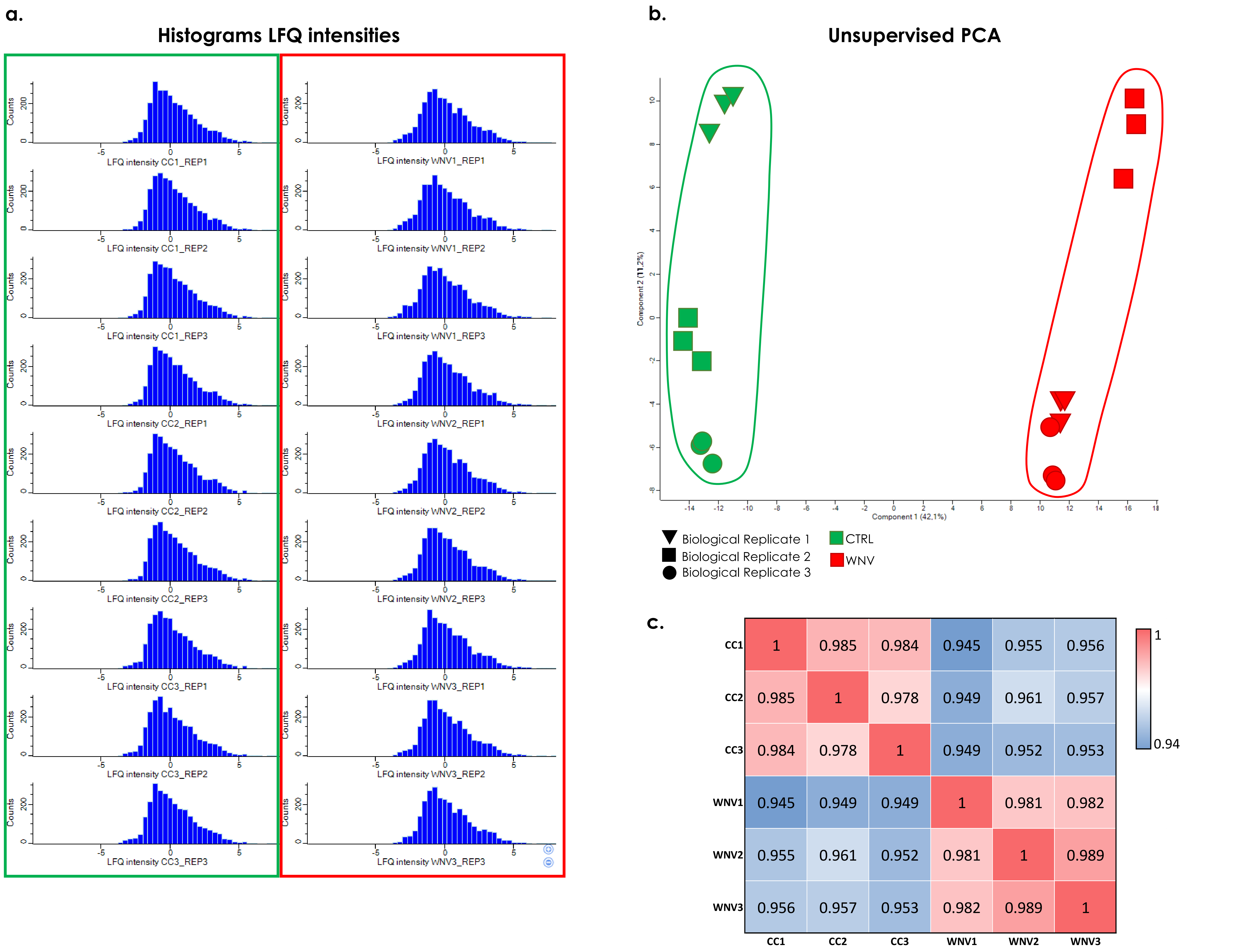

Figure S1. Quality control of Label-free quantitative LC-MS/MS proteomic data. **a)** histograms of LFQ intensities of each samples' LC-MS/MS run, specifically three technical replicates per each of the three biological replicate per CTRL (green) and WNV (red) condition. Runs are reproducible and robust with a normal distribution; **b)** Principal component analysis (PCA) of all quantifiable identified proteins. Each biological replicate is represented by different shapes, and green color indicates CTRL samples, while red color indicates WNV samples. PC1 summarizes 42,1% of variance, PC2 summarizes 11,2% or variance. **c)** Matrix correlation by Pearson's coefficient of LFQ values. Values equal to 1 indicate total linear positive correlation.

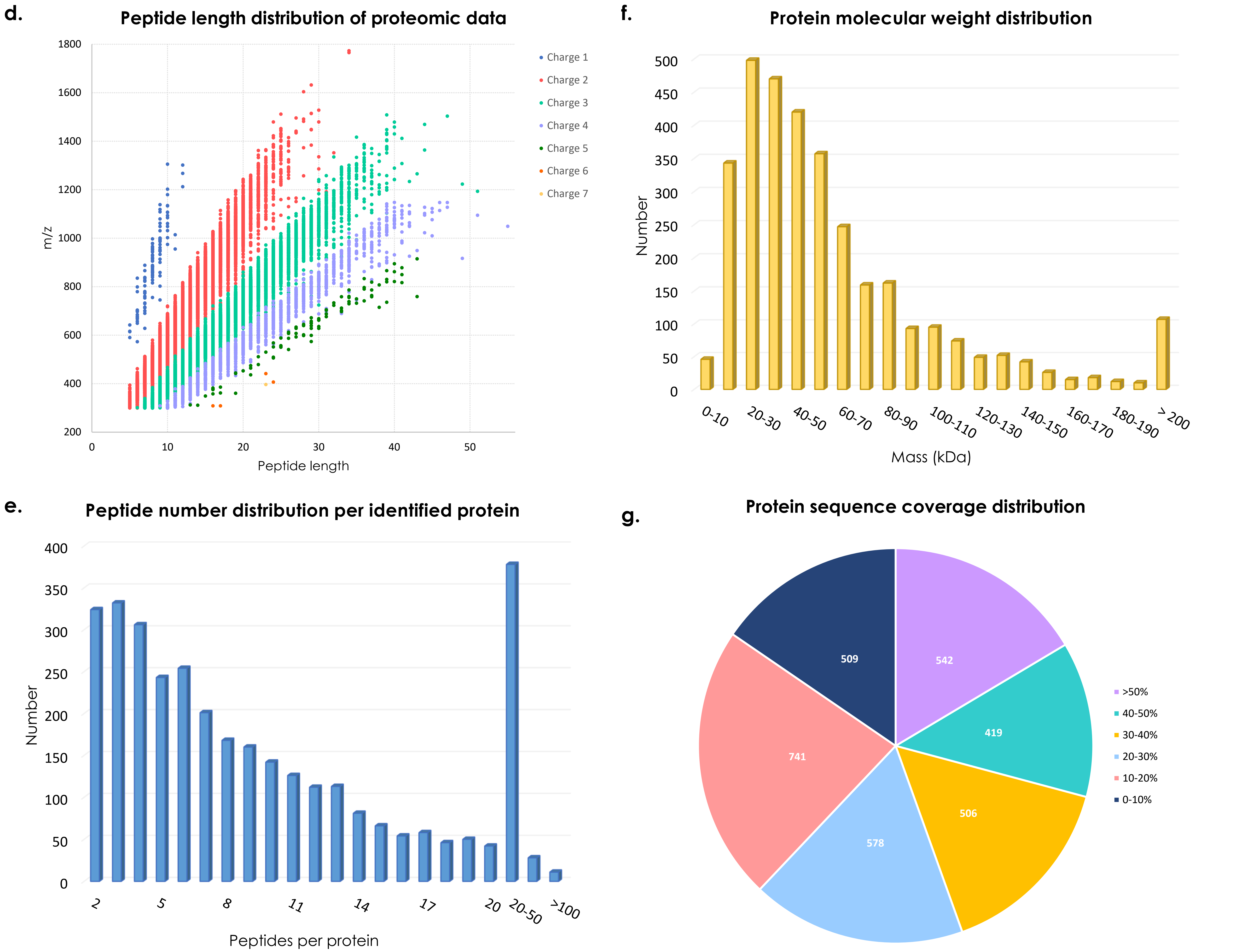

Figure S1. Quality control of Label-free quantitative LC-MS/MS proteomic data. d) Peptide length distribution, within optimal range of 7-20 amino acids; **e)** number distribution per identified protein, with most proteins identified with more than two peptides; **f)** protein molecular weight distribution; **g)** protein sequence converage distribution

	Sequence	Residues	Protein
	10 20 30 40 50 60 70 80 90 100		
	MSKKPGGPGK SRAVNMLKRG MPRVLSLIGL KRAMLSLIDG KGPIRFVLAL LAFFRFTAIA PTRAVLDRWR GVNKQTAMKH LLSFKKELGT LTSAINRRSS	1-105aa	Capsid protein C
100	KQKKR		
	GGKTG IAVMIGLIAS VGA		
	VTLSNFQ GKVMMTVNAT DVTDVITIPT AAGKNLCIVR AMDVGYMCDD TITYECPVLS AGNDPEDIDC WCTKSAVYVR	124-290aa	Protein prM
200	YGRCTKTRHS RRSRRSLTVQ THGESTLANK KGAWMDSTKA TRYLVKTESW ILRNPGYALV AAVIGWMLGS NTMQRVVFVV LLLLVAPAYS		
	FNCLGMSNRD		
300	FLEGVSGATW VDLVLEGDSC VTIMSKDKPT IDVKMMNMEA ANLAEVRSYC YLATVSDLST KAACPTMGEA HNDKRADPAF VCRQGVVDRG WGNGCGLFGK		
400	GSIDTCAKFA CSTKAIGRTI LKENIKYEVA IFVHGPTTVE SHGNYSTQVG ATQAGRFSIT PAAPSYTLKL GEYGEVTVDC EPRSGIDTNA YYVMTVGTKT	291-791aa	Envelope protein E
500	FLVHREWFMD LNLPWSSAGS TVWRNRETLM EFEEPHATKQ SVIALGSQEG ALHQALAGAI PVEFSSNTVK LTSGHLKCRV KMEKLQLKGT TYGVCSKAFK		
600	FLGTPADTGH GTVVLELQYT GTDGPCKVPI SSVASLNDLT PVGRLVTVNP FVSVATANAK VLIELEPPFG DSYIVVGRGE QQINHHWHKS GSSIGKAFTT		
700	TLKGAQRLAA LGDTAWDFGS VGGVFTSVGK AVHQVFGGAF RSLFGGMSWI TQGLLGALLL WMGINARDRS IALTFLAVGG VLLFLSVNVH A		
	DTGCAIDIS		
800	RQELRCGSGV FIHNDVEAWM DRYKYYPETP QGLAKIIQKA HKEGVCGLRS VSRLEHQMWE AVKDELNTLL KENGVDLSVV VEKQEGMYKS APKRLTATTE	792-1143aa	Non-structural protein 1 NS1
900	KLEIGWKAWG KSILFAPELA NNTFVVDGPE TKECPTQNRA WNSLEVEDFG FGLTSTRMFL KVRESNTTEC DSKIIGTAVK NNLAIHSDLS YWIESRLNDT		
1000	WKLERAVLGE VKSCTWPETH TLWGDGILES DLIIPVTLAG PRSNHNRRPG YKTQNQGPWD EGRVEIDFDY CPGTTVTLSE SCGHRGPATR TTTESGKLIT		
1100	DWCCRSCTLP PLRYQTDSGC WYGMEIRPQR HDEKTLVQSQ VNA		
	YNADMID PFQLGLLVVF LATQEVLRKR WTAKISMPAI LIALLVLVFG GITYTDVLRY		
1200	VILVGAAFAE SNSGGDVVHL ALMATFKIQP VFMVASFLKA RWTNQENILL MLAAVFFQMA YHDARQILLW EIPDVLNSLA VAWMILRAIT FTTTSNVVVP	1144-1374aa	Non-structural protein 2A NS2A
1300	LLALLTPGLR CLNLDVYRIL LLMVGIGSLI REKRSAAAKK KGASLLCLAL ASTGLFNPMI LAAGLIACDP NRKR		
	GWPATE VMTAVGLMFA IVGGLAELDI	1375-1505aa	Serine protease subunit NS2B
1400	DSMAIPMTIA GLMFAAFVIS GKSTDMWIER TADISWESDA EITGSSERVD VRLDDDGNFQ LMNDPGAPWK IWMLRMVCLA ISAYTPWAIL PSVVGFWITL		
1500	QYTKR		
	GGVLW DTPSPKEYKK GDTTTGVYRI MTRGLLGSYQ AGAGVMVEGV FHTLWHTTKG AALMSGEGRL DPYWGSVKED RLCYGGPWKL QHKWNGQDEV		
1600	QMIVVEPGKN VKNVQTKPGV FKTPEGEIGA VTLDFPTGTS GSPIVDKNGD VIGLYGNGVI MPNGSYISAI VQGERMDEPI PAGFEPEMLR KKQITVLDLH		
1700	PGAGKTRRIL PQIIKEAINR RLRTAVLAPT RVVAAEMAEA LRGLPIRYQT SAVPREHNGN EIVDVMCHAT LTHRLMSPHR VPNYNLFVMD EAHFTDPASI		
1800	AARGYISTKV ELGEAAAIFM TATPPGTSDP FPESNSPISD LQTEIPDRAW NSGYEWITEY TGKTVWFVPS VKMGNEIALC LQRAGKKVVQ LNRKSYETEY	1506-2124aa	Serine protease/helicase NS3
1900	PKCKNDDWDF VITTDISEMG ANFKASRVID SRKSVKPTII TEGEGRVILG EPSAVTAASA AQRRGRIGRN PSQVGDEYCY GGHTNEDDSN FAHWTEARIM		
2000	LDNINMPNGL IAQFYQPERE KVYTMDGEYR LRGEERKNFL ELLRTADLPV WLAYKVAAAG VSYHDRRWCF DGPRTNTILE DNNEVEVITK LGERKILRPR		
2100	WIDARVYSDH QALKAFKDFA SGKR		
	SQIGLI EVLGKMPEHF MGKTWEALDT MYVVATAEKG GRAHRMALEE LPDALQTIAL IALLSVMTMG VFFLLMQRKG	2125-2250aa	Non-structural protein 4A
2200	IGKIGLGGAV LGVATFFCWM AEVPGTKIAG MLLLSLLLMI VLIPEPEKQR		
	SQTDNQLAVF LICVMTLVSA VAA		
	NEMGWLD KTKSDISSLF GQRIEVKENF		
2300	SMGEFLLDLR PATAWSLYAV TTAVLTPLLK HLITSDYINT SLTSINVQAS ALFTLARGFP FVDVGVSALL LAAGCWGQVT LTVTVTAATL LFCHYAYMVP	2274-2528aa	Non-structural protein 4B
2400	GWQAEAMRSA QRRTAAGIMK NAVVDGIVAT DVPELERTTP IMQKKVGQIM LILVSLAAVV VNPSVKTVRE AGILITAAAV TLWENGASSV WNATTAIGLC		
2500	HIMRGGWLSC LSITWTLIKN MEKPGLKR		
	GG AKGRTLGEVW KERLNQMTKE EFTRYRKEAI IEVDRSAAKH ARKEGNVTGG HPVSRGTAKL RWLVERRFLE		
2600	PVGKVIDLGC GRGGWCYYMA TQKRVQEVRG YTKGGPGHEE PQLVQSYGWN IVTMKSGVDV FYRPSECCDT LLCDIGESSS SAEVEEHRTI RVLEMVEDWL		
2700	HRGPREFCVK VLCPYMPKVI EKMELLQRRY GGGLVRNPLS RNSTHEMYWV SRASGNVVHS VNMTSQVLLG RMEKRTWKGP QYEEDVNLGS GTRAVGKPLL		
2800	NSDTSKIKNR IERLRREYSS TWHHDENHPY RTWNYHGSYD VKPTGSASSL VNGVVRLLSK PWDTITNVTT MAMTDTTPFG QQRVFKEKVD TKAPEPPEGV		
2900	KYVLNETTNW LWAFLAREKR PRMCSREEFI RKVNSNAALG AMFEEQNQWR SAREAVEDPK FWEMVDEERE AHLRGECHTC IYNMMGKREK KPGEFGKAKG		
3000	SRAIWFMWLG ARFLEFEALG FLNEDHWLGR KNSGGGVEGL GLQKLGYILR EVGTRPGGKI YADDTAGWDT RITRADLENE AKVLELLDGE HRRLARAIIE	2529-3433aa	RNA-directed RNA polymerase NS5
3100	LTYRHKVVKV MRPAADGRTV MDVISREDQR GSGQVVTYAL NTFTNLAVQL VRMMEGEGVI GPDDVEKLTK GKGPKVRTWL FENGEERLSR MAVSGDDCVV		
3200	KPLDDRFATS LHFLNAMSKV RKDIQEWKPS TGWYDWQQVP FCSNHFTELI MKDGRTLVVP CRGQDELVGR ARISPGAGWN VRDTACLAKS YAQMWLLLYF		
3300	HRRDLRLMAN AICSAVPVNW VPTGRTTWSI HAGGEWMTTE DMLEVWNRVW IEENEWMEDK TPVEKWSDVP YSGKREDIWC GSLIGTRARA TWAENIQVAI		
3400	NQVRAIIGDE KYVDYMSSLK RYEDTTLVED TVL		
	3433		

Figure S2. WNV viral polyprotein identification by LC-MS/MS. Polyprotein sequence of viral WNV genome (3433 aa) encoding for 10 viral proteins. Red sequences correspond to sequenced peptides by LC-MS/MS analysis.
