# Supplementary material for: Extensive Proteomic Approach to Assess Protein Alterations in Human Hepatoma Huh7 Cells Infected with West Nile Virus
Source: Int J Mol Sci. 2026 Oct 21;27(20):9387. doi: 10.3390/ijms27209387 (PMC13705241; doi:10.3390/ijms27209387)
Supplement: Supplementary file 1 [file ijms-27-09387-s001.zip › Table S4.pdf]

Virus-Host PPI P-HIPSTer					
Top-down			Bottom-up		
Term	p-value	Genes	Term	p-value	Genes
West Nile virus lineage 2 envelope protein E (gene: flavivirus polyprotein)	1.17E-08	YWHAE;HSPA8;HSP90AA1;HSP90AB1;PSMD4;PSME3;GRB2	West Nile virus H442 non-structural protein 3	3.05E-02	AMBP;FN1
West Nile virus SA381/00 envelope protein	2.36E-08	YWHAE;HSPA8;HSP90AA1;HSP90AB1;PSMD4;PSME3;GRB2	West Nile virus SA381/00 non-structural protein 3	3.05E-02	AMBP;FN1
West Nile virus H442 envelope protein	3.12E-08	YWHAE;HSPA8;HSP90AA1;HSP90AB1;PSMD4;PSME3;GRB2	West Nile virus SA93/01 non-structural protein 3	3.05E-02	AMBP;FN1
West Nile virus lineage 2 excised_polyprotein 1..791 (gene: flavivirus polyprotein)	3.70E-07	YWHAE;HSPA8;HSP90AA1;HSP90AB1;PSMD4;PSME3;GRB2	West Nile virus SPU116/89 non-structural protein 3	3.05E-02	AMBP;FN1
West Nile virus SA93/01 envelope protein	4.91E-07	YWHAE;HSPA8;HSP90AA1;HSP90AB1;PSME3;GRB2	West Nile virus lineage 1 nonstructural protein NS3 (gene: flavivirus polyprotein)	3.05E-02	AMBP;FN1
West Nile virus SPU116/89 envelope protein	4.91E-07	YWHAE;HSPA8;HSP90AA1;HSP90AB1;PSME3;GRB2	West Nile virus lineage 2 nonstructural protein NS3 (gene: flavivirus polyprotein)	3.45E-02	AMBP;FN1
West Nile virus lineage 1 envelope protein E (gene: flavivirus polyprotein)	5.34E-07	YWHAE;HSPA8;HSP90AA1;HSP90AB1;PSME3;GRB2			
West Nile virus lineage 2 excised_polyprotein 1..2273 (gene: flavivirus polyprotein)	9.81E-07	YWHAE;HSPA8;HSP90AA1;VCP;HSP90AB1;PSME3;GRB2			
West Nile virus lineage 1 excised_polyprotein 1..787 (gene: flavivirus polyprotein)	2.28E-06	YWHAE;HSPA8;HSP90AA1;HSP90AB1;PSME3;GRB2			

Table S4. Virus-Host protein interaction prediction. Bioinformatic analysis of differential proteins by top-down (blue) and bottom-up (orange) approaches by Pathogen-Host Interactome Prediction using structure similarity by EnrichR, indicating potential interactors of WNV proteins.
