# Supplementary material for: Extensive Proteomic Approach to Assess Protein Alterations in Human Hepatoma Huh7 Cells Infected with West Nile Virus
Source: Int J Mol Sci. 2026 Oct 21;27(20):9387. doi: 10.3390/ijms27209387 (PMC13705241; doi:10.3390/ijms27209387)
Supplement: Supplementary file 1 [file ijms-27-09387-s001.zip › Table_S1.pdf]

Cells seeded per well	MOI	Time post-infection	Ct 1	Ct 2	$TCID_{50}$/mL
1.5 x 10^5	0.01	24 h	26.02	33.79	
	0.05		24.66	25.85	
3 x 10^5	0.005	24 h	26.36	24.94	
	0.01		23.70	23.16	
1.5 x 10^5	0.01	48 h	21.20	23.14	
	0.05		23.85	22.82	
**3 x 10^5**	**0.005**	**48 h**	**20.93**	**23.97**	**8.04×10^5**
	0.01	48 h	20.90	21.67	
1.5 x 10^5	0.01	72 h	36.23		
	0.05	72 h	26.20		
3 x 10^5	0.005	72 h	21.29		1.26×10^4
	0.01	72 h	21.64		

Table S1: **Time point and MOI evaluation of Huh7-WNV infection.** Huh-7 cells were seeded at different densities and infected at the indicated multiplicities of infection (MOIs). Viral RNA was assessed in culture supernatants at 24, 48, and 72 h post-infection by real-time reverse transcription polymerase chain reaction (RT-qPCR). "Ct 1" and "Ct 2" values represent the cycle threshold obtained for samples deriving from different infected cell cultures. $TCID_{50}$/mL (50% Tissue Culture Infectious Dose) was calculated for each viral supernatant at specific conditions (MOI 0.005 and both 48/72 h infection period). The experimental conditions selected for subsequent proteomic analyses (3×10^5 cells/well, MOI 0.005, 48 h post-infection) are highlighted in bold.
